# A topological approach to positron emission particle tracking for finding multiple particles in high noise environments

**DOI:** 10.1038/s41598-025-97175-0

**Published:** 2025-04-19

**Authors:** Jack A. Sykes, Andrei L. Nicuşan, Dominik Werner, Matthew T. Herald, Daniel Weston, Tzany Kokalova Wheldon, Christopher R. K. Windows-Yule

**Affiliations:** 1 School of Physics and Astronomy, University of Birmingham, Edgbaston, Birmingham B15 2TT UK; 2 School of Chemical Engineering, University of Birmingham, Edgbaston, Birmingham B15 2TT UK

**Keywords:** Chemical engineering, Experimental nuclear physics, Computer science

## Abstract

Positron emission particle tracking (PEPT) is an advanced imaging technique that accurately tracks the three-dimensional spatial coordinates of a radioactively-labelled particle with sub-millimetre and sub-millisecond precision. By detecting back-to-back 511 keV gamma rays from positron-electron annihilation coincidence events, PEPT can locate particles within highly dense, opaque systems such as fluidised beds, rotating drums, and mills. Despite the progress made in enhancing the precision and accuracy of PEPT, simultaneous multiple particle tracking remains a significant challenge, particularly in high-noise environments. This paper introduces T-PEPT, a novel algorithm that leverages topological data analysis-a relatively new field of applied mathematics that explores the underlying ’shape’ of data through techniques like persistence homology. By creating simplicial complexes and applying persistence homology to PEPT point data, T-PEPT demonstrates highly effective performance in multiple-particle tracking, especially in scenarios with high noise. When benchmarked against existing PEPT algorithms using a widely recognised standard framework, T-PEPT consistently maintains sub-millimetre spatial and sub-millisecond temporal precision in nearly all cases, demonstrating its robustness and accuracy. For Data availability for T-PEPT, please use the GitHub repository: https://github.com/uob-positron-imaging-centre/pept.

## Introduction

Positron emission particle tracking (PEPT) is a Lagrangian technique that tracks the three-dimensional spatial coordinates of a radioactively-labelled particle within complex systems such as rotating drums^1^, fluidised beds^2^, and stirred tanks^3^. By detecting back-to-back 511 keV gamma rays from positron-electron annihilation coincidence events in a positron emission tomography (PET) detector, PEPT achieves high spatial and temporal resolution, making it suitable for tracking particles in dense, opaque systems where optical methods, such as particle tracking velocimetry (PTV), struggle^4^. However, PEPT faces challenges, particularly in simultaneous multiple particle tracking and high-noise environments, where noise and false coincidences complicate the accurate determination of particle trajectories^5,6^.

Although there is no single, accepted of what represents a high-noise environment, for the present paper we take the definition to be when signal and noise become comparable. Applying this to PEPT, high-noise is when the ratio of “true” to “false” coincidence events is equal or small. Noise increases when the number of true, uncorrupted gamma-ray pairs becomes smaller than the number of false coincidences, scattered pairs, or background radiation. Here, false coincidences refer to pairs formed by unrelated photons from separate annihilations, while scattered pairs involve one or both photons undergoing Compton scattering within the system or detector, reducing spatial resolution. Both are considered ‘corrupted’ events in this work, as they degrade tracking accuracy through distinct physical processes. This noise is increased by materials with high density and scatter properties such as steel, see Experimental Simulations section, which significantly reduce true pair detections and increase scattered or corrupted pair detections - see Windows-Yule et al.^7^ for more detail on false pair detections.

Various algorithms have been developed to enhance PEPT’s precision and accuracy since the development of the first PEPT algorithm, the Birmingham Method^5^, including PEPT using Machine Learning (PEPT-ML)^8^ and an expectation-maximization algorithm (PEPT-EM)^9^. The Birmingham Method is known for its simplicity and robustness in single-particle tracking but struggles with multiple-particle tracking in high-noise environments, where distinguishing between true and false coincidences is critical. PEPT-ML, PEPT-EM, and other PEPT algorithms address some of these challenges by applying advanced clustering techniques to better distinguish multiple particles but overall still find high-noise, multiple tracer scenarios challenging^10^.

Topology, specifically algebraic topology, offers new tools for addressing these challenges. Topological Data Analysis (TDA) leverages these principles to analyse the shape of data, using methods like persistent homology to identify significant features across multiple scales^11,12^. TDA has become a powerful tool for analysing complex datasets, especially where traditional metric-based approaches fall short. This paper introduces T-PEPT, a novel algorithm that applies TDA to improve multiple-particle tracking, particularly in high-noise environments. By constructing simplicial complexes and applying persistent homology to PEPT point data^13,14^, T-PEPT captures the underlying structure of particle trajectories, identifying persistent features corresponding to true particle paths while filtering out noise. T-PEPT is scalable and adaptable, capable of handling varying levels of complexity within tracked systems, from simple single-particle environments to highly dynamic multiphase systems.

This paper covers the theoretical foundations of topology, provides an overview of PEPT and its algorithms, introduces the new T-PEPT algorithm and demonstrates its application in a multiple-particle, high-noise experiment. Specifically, this case study focuses on detecting multiple tracers moving in a densely-packed, rotating attritor mill with attenuating materials to simulate a high-noise experiment. By resolving the dynamic trajectories of multiple particles in a high-noise environment, we are ensuring the robustness and versatility of T-PEPT is tested. Further details on the topological methods used, and a comprehensive comparison of T-PEPT against contemporary PEPT algorithms, are provided in the Supplementary Material. For a deeper understanding of PEPT and its various algorithms, see works by Parker et al.^5,6,15^ and Windows-Yule et al.^10,16^. For more on topological methods, see Carlsson^13^ and Chazal et al.^11^.

## Methods

### Topological data analysis

Topology is a branch of mathematics that studies the continuity and connectivity of intrinsic geometric properties of higher-dimensional surfaces and manifolds within a space, including the shape, the number of holes, and connected components. In other words, topology is the study of qualitative geometric information^13,17,18^. The field has significantly advanced over the years, covering an extensive array of topics such as knot and graph theory, topological optimisation, topological data analysis, and topological materials and devices^13,18,19^. Modern applications of topology span numerous fields. In computer science, topology aids in data analysis and network theory, such as analysing the spread of epidemics^20^. In physics, topology contributes to understanding the properties of exotic states of matter^21^, while in biology, it assists in studying the shapes and folding patterns of molecules^22^.

**Fig. 1:**
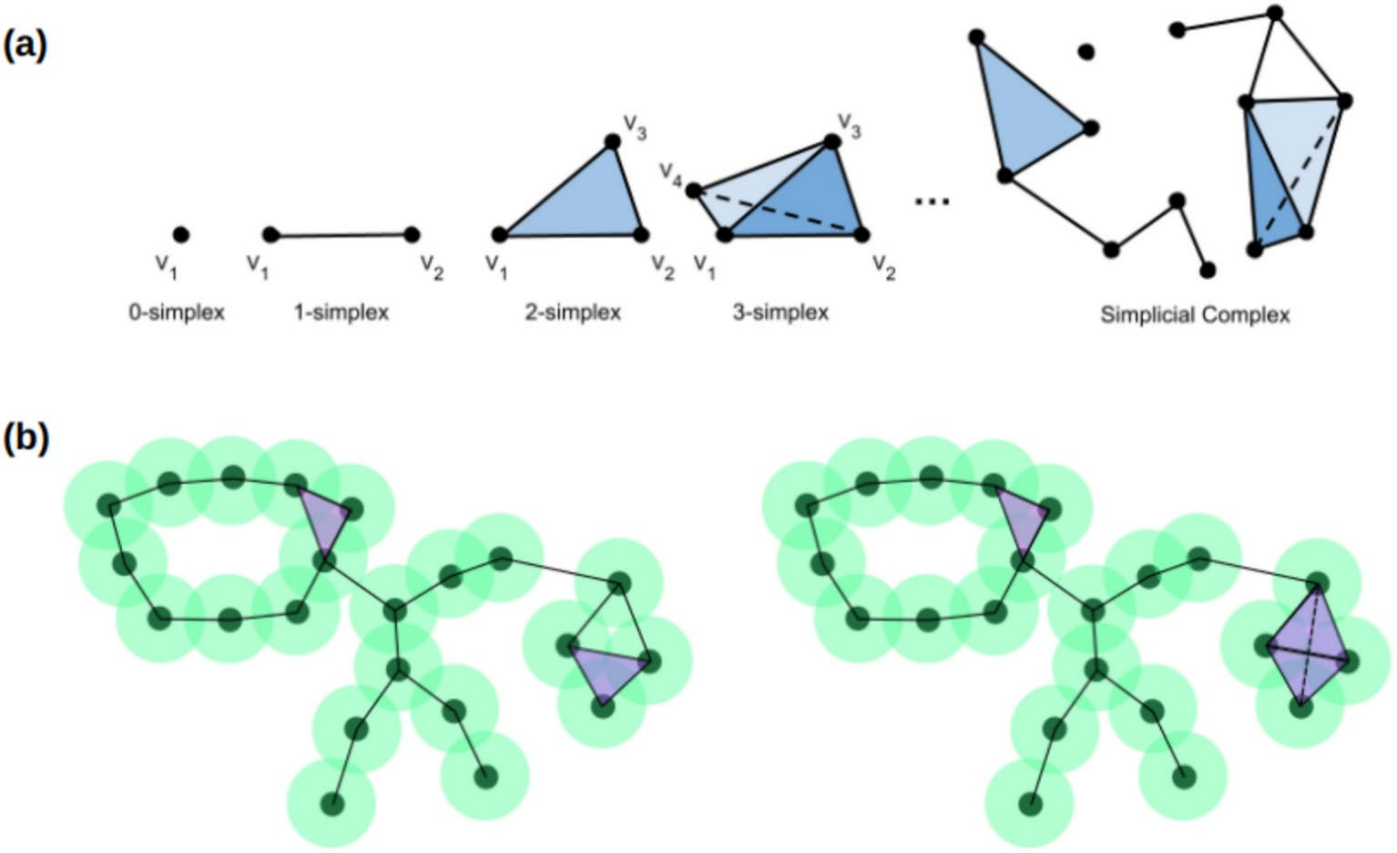
(**a**) From left to right: a vertex (0-simplex), a line (1-simplex), a triangle (2-simplex), a tetrahedron (3-simplex), and so on into higher dimensions. The graph on the right is an example of a simplicial complex with three disjoint connected sets. (**b**) Čech complex (left) versus Vietoris-Rips complex (right). The Čech complex forms loops by requiring all vertices to share a common intersection, while the V-R complex connects vertices pairwise, leading to the formation of higher-dimensional shapes.

A fundamental concept in topology is the simplicial complex, which is constructed from simplices-geometric objects like points (0-simplices), line segments (1-simplices), triangles (2-simplices), and their higher-dimensional counterparts (see Fig. 1a). These complexes provide a framework for understanding the underlying structure of data by examining how these simplices are connected^11,13^. In the context of PEPT, a simplicial complex can represent clusters of reconstructed particle positions, where points correspond to cutpoints derived from gamma-ray pairs, and edges or higher-order simplices connect nearby points within a threshold distance. Simplicial complexes can be constructed through the concept of “closeness” of points, which may be determined by Euclidean distance - as with the case for T-PEPT - or any other relevant metric, depending on the application.

Two types of simplicial complexes often used are the Čech complex and the Vietoris-Rips (V-R) complex. A common way to construct these complexes is to create a Euclidean circle (or sphere in three dimensions) around every point in a dataset. As the radii of these circles or spheres increase, they begin to overlap or intersect; when they do, a line connects the intersecting points. The Čech complex forms simplices by requiring all vertices to share a common intersection point, which can be computationally expensive in higher-dimensional spaces. Conversely, the V-R complex is constructed by connecting vertices pairwise, leading to the formation of higher-dimensional shapes, and is computationally more feasible for large datasets (see Fig. 1b)^11^. The V-R complex was selected for T-PEPT for this reason, in which dynamic particle trajectories can be tracked in high-noise environments whilst minimising computational expense.

**Fig. 2:**
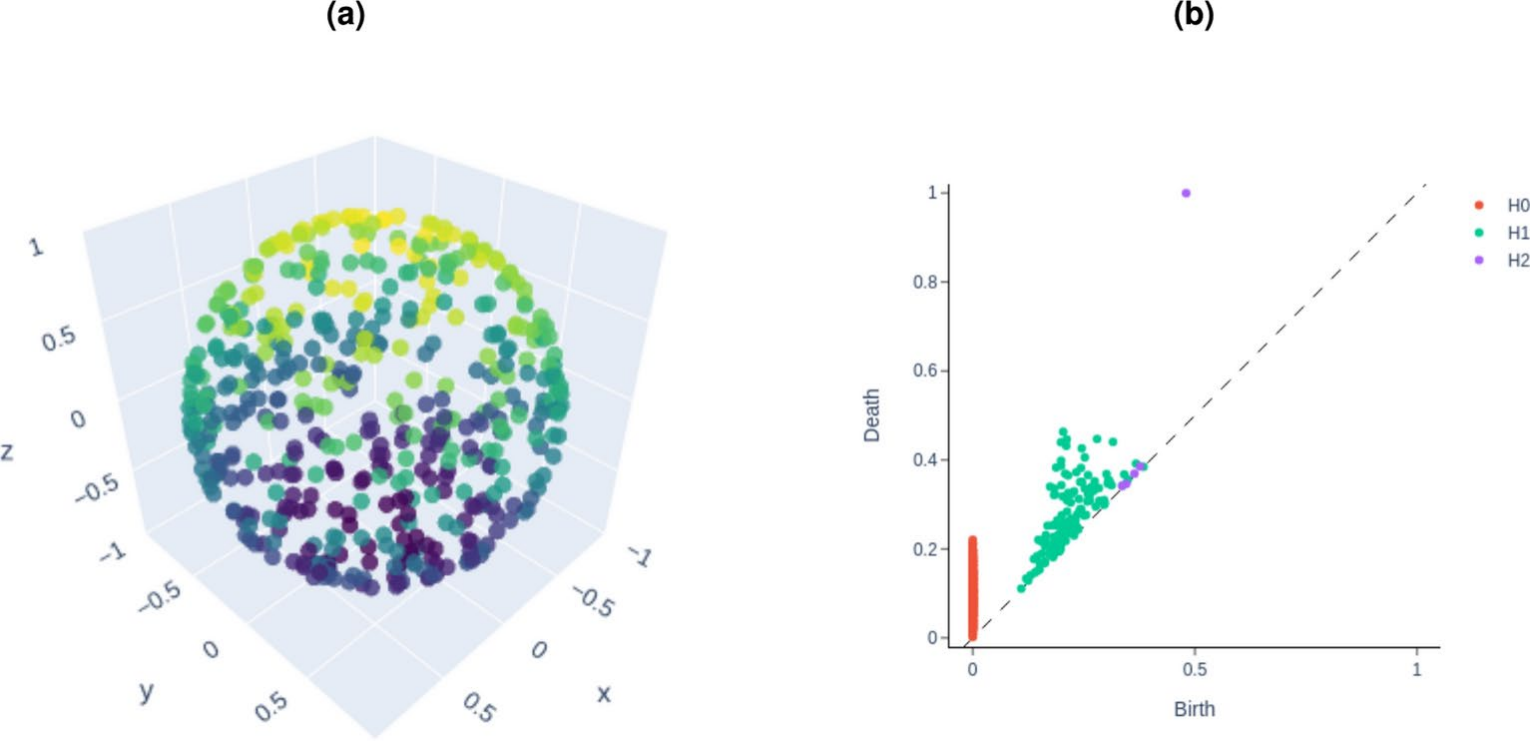
(**a**) A hollow sphere point cloud comprised of 500 points. (**b**) The corresponding persistence diagram showing homology groups H$_{0}$ (connected components like points or edges), H$_{1}$ (loops formed within data), and H$_{2}$ (holes or voids in the data). As we expect, the hollow sphere has one persisting hole in the dataset, visualised by the outlying H$_{2}$ point. The colouring in (**a**) simply represents the Z-position of the data points, allowing for easier visualisation of the three-dimensional plot.

Persistent homology is a tool in algebraic topology that computes the topological features of a dataset and studies them across a range of spatial resolutions. Robust features that persist over a large interval are considered significant, while less persistent features are often deemed noise^14,23^. A ‘filtration’ process is applied to evaluate the importance of topological properties. The filtration ranks and subsequently removes topological attributes based on their increasing importance, with the removed attributes considered as noise^14^. Persistent homology is most commonly visualised using a birth-death persistence diagram, where each point represents a topological feature, and its coordinates indicate the scale at which the feature appears and disappears - see Fig. 2 for an example. In PEPT, persistent features correspond to true particle locations, while less persistent features represent noise from scattered or false gamma-pair detections. Fig. 4 in Section 2.4 of the Supplementary material shows persistent homology applied to real PEPT data and the corresponding birth-death diagram.

TDA employs these methods to analyse the shape of data by restructuring it into a family of simplicial complexes and applying persistent homology to the set. TDA is particularly robust against noise because it focuses on the shape and patterns within the data rather than the distance between points and other geometric properties. In high-dimensional spaces, where traditional analysis can be challenging due to the “curse of dimensionality”, TDA’s ability to focus on global structures rather than individual dimensions provides a significant advantage^11,13,24^.

The Topological Mode Analysis Tool (ToMATo) is a clustering algorithm that combines topological persistence with traditional clustering methods, allowing for the identification of significant clusters that persist across a wide range of scales. ToMATo is especially effective in ensuring that only the most topologically significant features are retained, which makes it particularly useful for analysing complex data structures^25^. T-PEPT takes inspiration from the principles used in ToMATo and other TDA methods, with further detailed explanations and additional figures related to these topological methods provided in the Supplementary Material Section 1. T-PEPT leverages these principles to dynamically cluster PEPT data - by applying TDA, T-PEPT distinguishes persistent structures (true tracer locations) from noise, even in high-noise environments such as mills^26^.

### Topological positron emission particle tracking (T-PEPT)

**Fig. 3:**
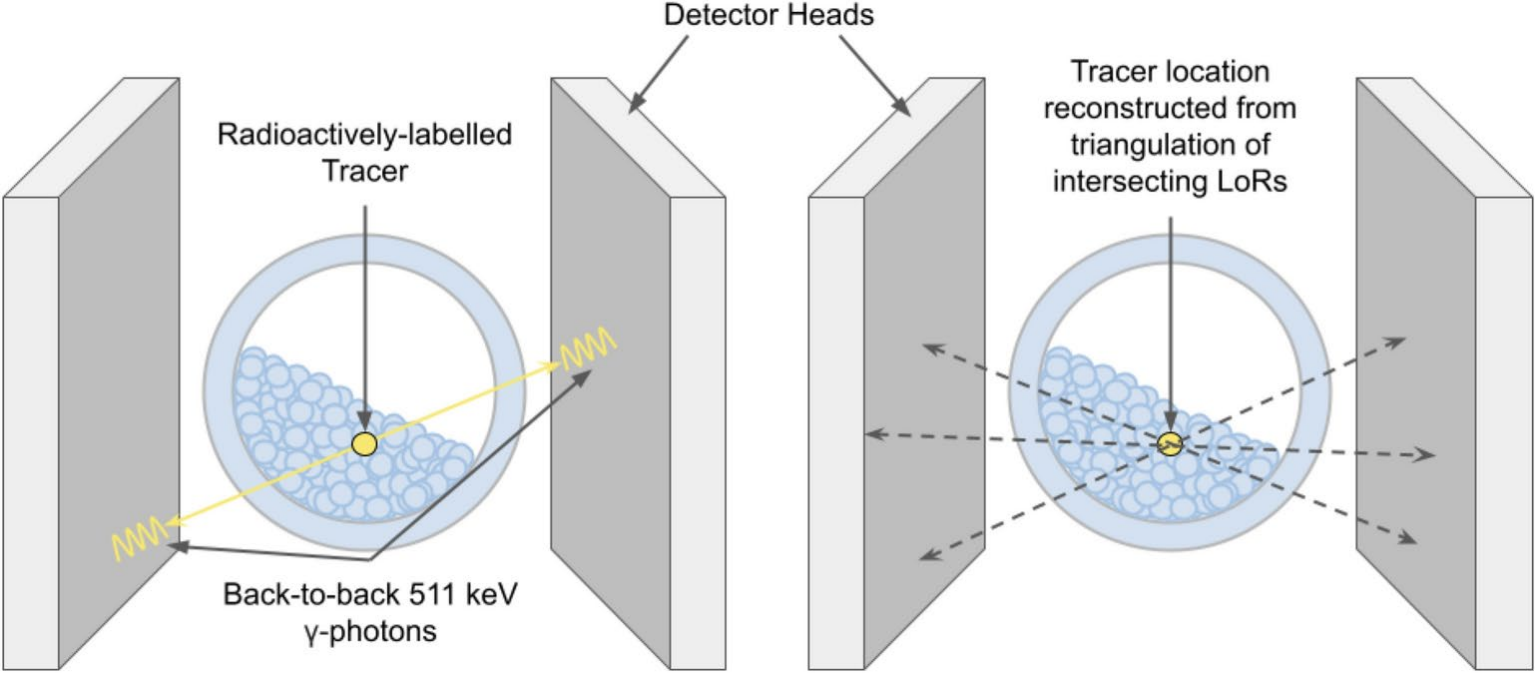
A schematic of the principles of PEPT using the Philips ADAC Forte dual-headed positron detector: the intersection of LORs are used to determine the position of the positron-emitting tracer in a dense granular system, such as a rotating drum. This figure is adapted from Windows-Yule et al., Annual Review of Chemical and Biomolecular Engineering, Vol 11, Positron Emission Particle Tracking of Granular Flows, Copyright Annual Reviews (2020)^16^, CC BY open access license.

In the PEPT process, a particle, such as a glass bead or ion-exchange resin, is taken from a system of interest, labelled with a positron-emitting isotope such as fluorine-18 (F-18), and returned to the system. The positrons emitted from the tracer annihilate with electrons very close to the tracer’s location (within 2 mm in air and shorter distances in, say, water^27^), producing two back-to-back 511 keV photons. The line between two detected 511 keV gamma-rays is known as the Line-of-Response (LOR); when detected in coincidence, a sample size of $N_{LORs}$ = 100 - 250 LORs (but can be as low as 10 or much higher if there is a significant amount of noise) are triangulated to determine the position of the tracer at a given timestamp^5,15,16^. This process can be seen in Fig. 3 using the Philips ADAC Forte detector as an example. Consecutive tracer positions are clustered to calculate the trajectory of the tracer. However, in reality, a significant number of ‘false’ coincidence events result in false LORs being detected. Random secondary and background photons within an energy threshold and scattered gamma rays can be detected and need to be removed to identify the ‘true’ LORs^6^. The final stage of PEPT is to post-process the data in the form of calculating velocity fields, velocity distributions, occupancy plots, and similar processes relevant to the analysis of the system, such as fluidised beds, rotating drums, mixers, and pipe flows^16,28–31^. PEPT has also been used to image more modern systems such as continuous blenders^32^ and even to develop the first radiolabelled sub-micrometre living cell for biomedical PEPT^33^.

Many distinct algorithms have been developed for PEPT, tailored for different types of detectors and configurations. The Birmingham Method, the original PEPT algorithm developed at the University of Birmingham by David Parker and Michael Hawkesworth, remains the most widely used to date^5^. To track a single tracer, the algorithm calculates a first approximation Minimum Distance Point (MDP) between all trajectories of LORs within a sample, N, rejecting those furthest away and recalculating the MDP iteratively until a fixed, predetermined fraction of the original coincidence events, f, is left. During this process, corrupt coincidence events produced by Compton scattered gamma rays and random pairs of 511 keV gamma rays unrelated to the annihilation coincidence event are removed. The remaining fN LORs determine the tracer’s position, with the algorithm providing the location of the annihilation coincidence event and the timestamp at which it occurred, outputting files in the format of time and three-dimensional spatial coordinates: (t, x, y, z)^5,6,15^. Since its development, the Birmingham Method has been adapted for multiple-particle tracking by iteratively repeating the process for each tracer. Initially, discarded coincidence events are reprocessed to find subsequent tracer locations, with the particle exhibiting the highest data logging rate chosen first, and so on as the algorithm iterates^8,34,35^. Further developments of the Birmingham Method include dynamic parameter optimisation, which reduces spatial errors over the default parameters^36^.

One of the more recently developed algorithms is PEPT-ML, created by Nicuşan and Windows-Yule^8^, which utilises machine learning in a two-pass clustering sequence to track multiple particle trajectories based on their LORs in Euclidean space. The algorithm addresses shortcomings in other PEPT algorithms, such as the need for *a priori* knowledge of the number of tracers in the field of view FOV and the challenge of maintaining accuracy and acquisition rates as the number of particles increases^8,10^. The key steps of PEPT-ML are: (1) splitting data into “samples” of LORs; (2) calculating the point (known as a “cutpoint”) that minimises the distance between every pair of LORs; (3) clustering these cutpoints using the Hierarchical Density-Based Spatial Clustering of Applications with Noise (HDBSCAN)^37^ algorithm, then extracting the centres of the clusters (“1-pass clustering”); (4) splitting the cluster centres into new sample sizes; (5) repeating step 3 to obtain a new set of cluster centres (“2-pass clustering”); (6) constructing particle trajectories from the centres identified in step 5. PEPT-ML has demonstrated high spatial and temporal resolution, with the ability to distinguish multiple tracers by distances as small as 2 mm and to track up to 128 individual tracers, a limitation determined by the computational resources available to the authors^8^. Both the Birmingham Method and PEPT-ML were included in a benchmarking study of PEPT algorithms conducted by Windows-Yule et al.^10^ - see Supplementary Material Section 3.

For a comprehensive overview of how all PEPT algorithms operate and compare in terms of their advantages and disadvantages, refer to the works by Windows-Yule et al.^10^ and Nicuşan et al.^8^. Additionally, for an in-depth explanation of PEPT, including the different detector systems and tracers used, see the papers by Parker et al.^5,6,15^ and Windows-Yule et al.^7,10,16^.

This paper introduces the novel PEPT algorithm, T-PEPT, which integrates topological data analysis with clustering techniques and traditional PEPT methods to enhance radioactive particle tracking. Implemented into the PEPT library for direct comparison with other algorithms and freely available to the community (see here https://github.com/uob-positron-imaging-centre/pept), T-PEPT follows these key steps:

- *Data segmentation and cutpoints calculation*: PEPT data is segmented into samples, and for each sample, “cutpoints” are calculated by minimising the distance between pairs of LORs. This ensures accurate particle position determination with adjustable sample sizes to balance temporal and spatial resolution.
- *Topological clustering*: A Gaussian Kernel Density Estimation (KDE) is applied to cutpoints to identify high-density regions, assigning each point a filtration value f(p) = $\rho$(p), where $\rho$(p) represents the local density. Nearest neighbours are identified using a k-dimensional tree to construct a simplicial complex, with edges weighted by the average density of their endpoints f(e)= ($\rho$(p$_{1}$) + $\rho$(p$_{2}$)) / 2 (see Section 2.2 of the Supplementary Material for more details). The simplicial complex is analysed using a simplex tree, where filtration values prioritise dense clusters, making the approach robust to noise.
- *Noise-to-signal ratio calculation*: Filtration clusters are evaluated to distinguish between noise and significant data, dynamically calculating the “*true_fraction*” parameter (the ratio of signal-to-noise). Unlike the user-defined *true_fraction* used in the PEPT-ML algorithm, T-PEPT’s dynamic calculation adapts to the specific dataset, effectively eliminating the need for manual parameter tuning and ensuring adaptive refinement.
- *(Optional) Persistence diagram plotting*: Users can generate a persistence diagram (see Fig. 2b) using persistent homology to explore the topological features of the data. The diagram aids in visualising the stability of identified structures across scales.
- *Spatial clustering*: First-Pass Clustering: HDBSCAN is used to cluster cutpoints based on topological insights. An artificial noise cluster is introduced outside the system’s FOV to improve noise differentiation. The dynamically calculated *true_fraction* value is applied to adjust clustering sensitivity.
- *Second-pass clustering*: Centroids from the first pass are re-clustered to improve spatial accuracy. The *true_fraction* parameter is recalculated for each sample, enhancing the clustering of complex datasets.
- *Particle Trajectory Reconstruction*: Final cluster centroids are used to reconstruct particle trajectories. T-PEPT utilises the relationship between tracer activity and the number of cutpoints to maintain accurate trajectory tracking, even when tracers intersect or collide. The method also accounts for variations in PET detector sensitivity across the FOV.

Further details on T-PEPT can be found in the Supplementary Material Section 2.

### Experimental simulations

**Fig. 4:**
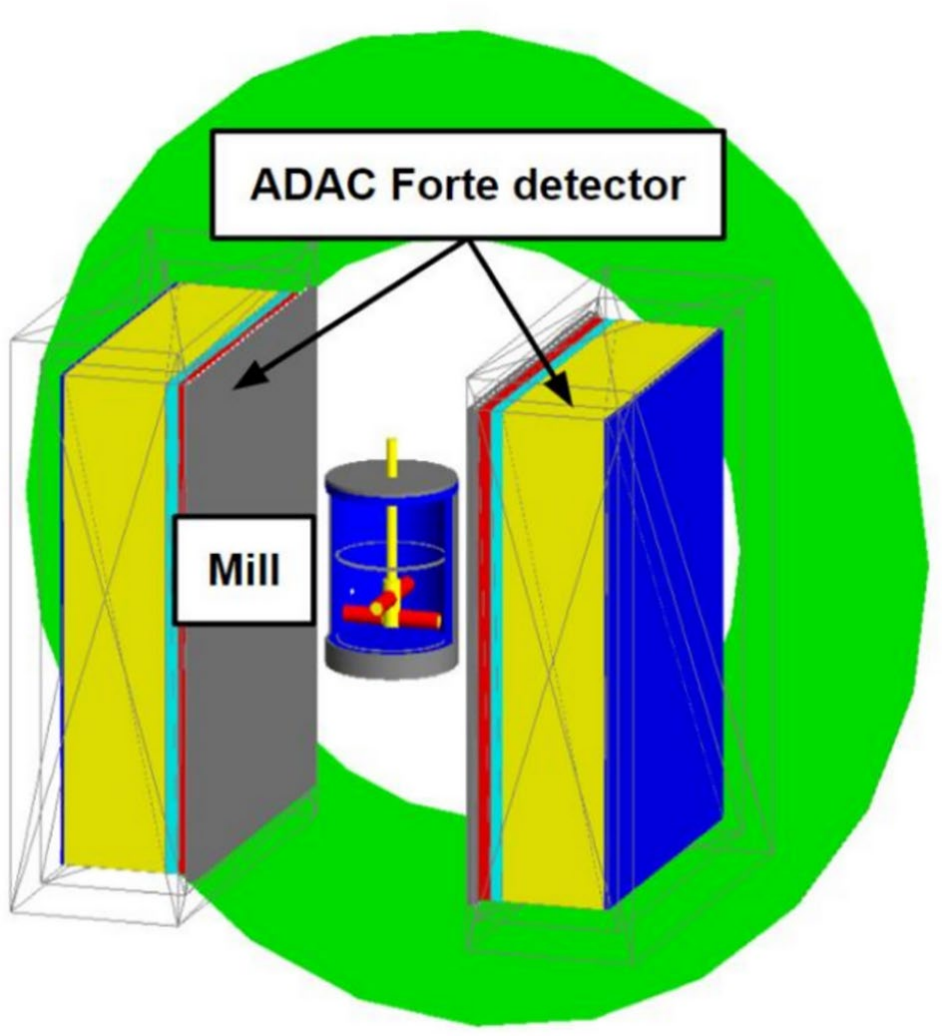
Visualisation of the attritor mill inside the Phillips ADAC Forte detector simulated in GATE. This figure was published in Nuclear Instruments and Methods in Physics Research Section A: Accelerators, Spectrometers, Detectors and Associated Equipment, Vol 1047, M. Herald et al. Improving the accuracy of PEPT algorithms through dynamic parameter optimisation, Copyright Elsevier (2023)^36^, CC BY open access license.

To test the challenging scenarios of multiple particle tracking in high-noise environments, simulations of a real-world industrial application was modelled using the discrete element method (DEM)^38^ and the Monte Carlo simulation method, Geant4 Application for Tomographic Emission (GATE)^39^. Specifically, an industrial attritor mill^26^ was chosen due to its widespread application across various industries, such as food^40^, pharmaceuticals^41^, and Fast Moving Consumer Goods (FMCG)^42^. Importantly, it represents an ideal test case for densely packed, rapidly moving, attenuating particles within a metal container” i.e. a multiple-particle, high-noise environment. The GATE model of the mill, positioned within the Phillips ADAC Forte detector, was adapted from the work of Herald et al.^36^, and a visualisation of this setup is shown in Fig. 4.

Numerous simulations were performed to explore a range of challenges for testing T-PEPT and comparing its performance against the Birmingham Method and PEPT-ML. Five sphere tracers were selected to represent particles, with their trajectories generated using a DEM simulation of the mill. The DEM software employed was LIGGGHTS^43^. PEPT has been used to validate DEM simulations in numerous cases^44^, including mills^45^. The mill features 10 mm thick walls, an impeller shaft with four pins, and was filled with 6 kg of steel spheres, each 5 mm in diameter, rotating at 300 RPM for 10 complete revolutions after achieving the target speed. This procedure was then repeated at 600 RPM. In both cases, the x, y, and z-positions of the particles were recorded at 0.05 second intervals - representative of real PEPT data from the Phillips ADAC Forte detector - and exported to CSV files, which were subsequently imported into the GATE simulation. The five tracers were assigned these CSV trajectories and labelled with 10 MBq of the radioisotope fluorine-18. The DEM simulation began with 11 s of ‘warm-up’ time: 5 s of filling the mill, 1 s for them to settle, and 5 s ramping up the RPM to the desired value.

GATE simulations were conducted at both 300 RPM and 600 RPM under three different conditions:

- Glass tracers with aluminium mill walls.
- Glass tracers with steel mill walls.
- Steel tracers with steel mill walls.

These conditions were chosen to evaluate tracking across varying noise levels, with the glass tracers in aluminium walls (density 2.70 gcm$^{-3}$ and linear attenuation coefficient 0.226 cm$^{-1}$)^10^ representing the least noisy scenario, and the steel tracers in steel walls (density 7.92 gcm$^{-3}$ and linear attenuation coefficient 0.659 cm$^{-1}$)^10^ representing the most noisy and therefore the most challenging scenario. Pre-computing the trajectories using the DEM simulation ensured accurate modelling of particle paths and collisions within the mill. Employing GATE subsequently allowed us to assign and label the five tracers with positron-emitting radiation, which was then used to assess the tracking performance of T-PEPT, the Birmingham Method, and PEPT-ML. Coincidence events were defined using a 350-650 keV energy window and a 15 ns time window, balancing detection efficiency while minimising false coincidences.

## Results

### Evaluation against standard testing framework

T-PEPT has been evaluated against other PEPT algorithms through a Standard Testing Framework for PEPT experiments, which assess both single and multiple particle tracking capabilities. These tests have been developed to allow for a fair, quantitative, and comprehensive comparison of the PEPT algorithms, assessing their strengths and weaknesses^10^. The tests are split into two parts: single and multiple particle tracking. The single particle tracking tests include minimum activity, maximum velocity, scatter sphere, and FOV experiments. The multiple particle tracking tests include minimum separation, false positive, orientation tracking, and large tracer number experiments. These tests cover a wide range of typical PEPT-like scenarios for two types of PEPT detectors: the Phillips ADAC Forte and Siemens ECAT EXACT HR+ detectors, both simulated in GATE. For more detail on each test, please read the paper by Windows-Yule et al.^10^. All relevant data and testing functions can be found on GitHub here https://github.com/mxh1092/RoPP-Comparison-Functions.

**Fig. 5:**
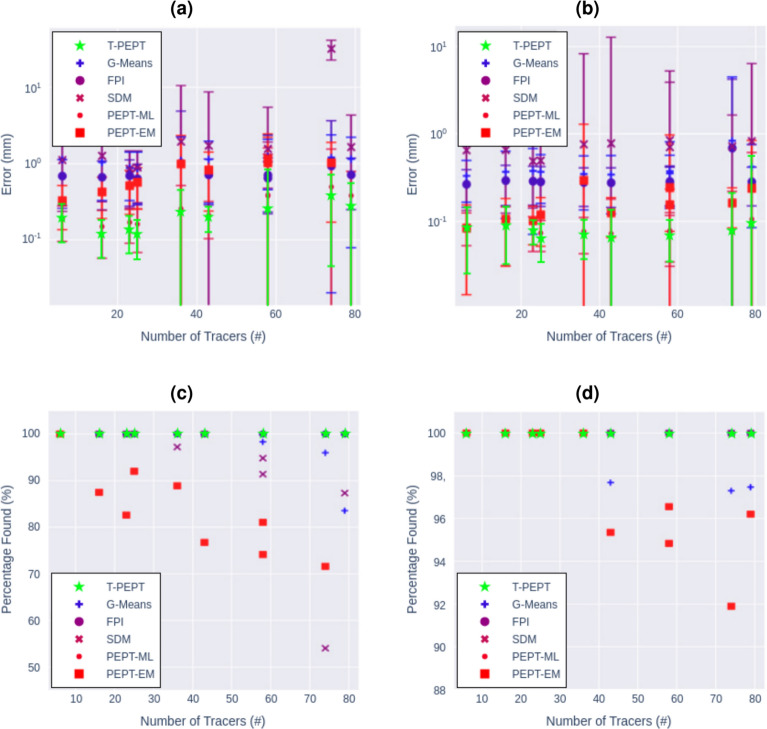
Above: the means error of the PEPT detected positions from the multiple particle tracking test compared to the nearest GATE prescribed particle positions (**a**) Forté geometry. (**b**) ECAT geometry. Below: the percentage of particles found by each PEPT algorithm compared to the number prescribed GATE particles. (**c**) Forté geometry. (**d**) ECAT geometry. The number of LORs per sample used for T-PEPT ranged from 500 to 12,500. This figure is adapted from Windows-Yule et al. Reports on Progress in Physics, Vol 85, Recent advances in positron emission particle tracking: a comparative review, Copyright IOP Publishing Ltd (2022)^10^, CC BY open access license.

T-PEPT performed among the best for the minimum activity, maximum velocity, minimum separation, false positive, and orientation tracking tests. T-PEPT slightly under-performed in the scatter sphere and FOV tests, and over-performed in the maximum tracers test. Full details of the results are provided in Section 3 of the Supplementary Material. Figure 5 shows the results of the maximum tracer test, where the algorithms were tasked to track an increasing number of randomly located tracers, ranging from 6 to 79, without *a priori* knowledge of the number. This test is arguably one of the most difficult tests.

### Evaluation in an attritor mill

**Fig. 6:**
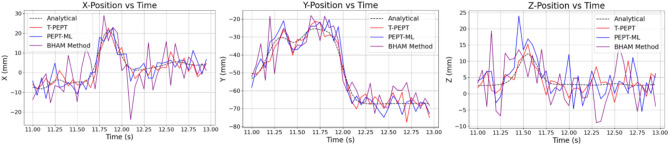
Comparison of the 3D spatial positions for T-PEPT (red), PEPT-ML (blue), and the Birmingham Method (purple) against the DEM-GATE (black dotted) analytical trajectory. Note this is for the 600 RPM, steel tracers and steel mill walls scenario, i.e. the most ‘noisy’and challenging environment, for 10 rotations for a single tracer. 1000 LORs per sample were used for each algorithm.

The primary objective of this study was to evaluate T-PEPT’s capability to track multiple moving tracers in high-noise environments within a simulated attritor mill. Fig. 6 presents the reconstructed spatial trajectories (X, Y, and Z positions) of a single tracer for the 600 RPM case, using steel tracers with steel mill walls-representing the most challenging scenario. The trajectories for T-PEPT, PEPT-ML, and the Birmingham Method are compared against the analytical DEM-GATE trajectory (shown as a dotted black line), where the true path of the tracer was predetermined.

Simultaneous tracking of all five tracers was performed, and the average errors in spatial positions (X, Y, and Z) for all five tracers are shown in Table 1. The standard deviation (STD) of these errors were also calculated. The STD provides an indication of the variability in the tracked particle positions for each technique. This procedure was repeated for three scenario types given in the Experimental Simulations section, as well as for the 300 RPM case. The largest errors occur in the Y-direction, corresponding to perpendicular to the detector heads, where the Phillips ADAC Forte has the least accurate spatial resolution^46^. For both RPM cases, T-PEPT successfully tracked all five tracers across all scenarios, whereas PEPT-ML and the Birmingham Method tracked only four and three tracers, respectively, in the highest-noise environment for both the 300 RPM and 600 RPM cases - for the 300 RPM data see Section 4 of the Supplementary Material.

**Table 1:** Average X-, Y-, and Z-spatial errors for T-PEPT, PEPT-ML, and the Birmingham Method for the 600 RPM case scenarios. Data for which PEPT-ML and the Birmingham Method were unable to locate all 5 tracers successfully are not considered. The number of LORs per sample was 1000 for all three algorithms.

Scenario	Method	No. tracers located	Error (mm)		
			X	Y	Z
Glass tracer, aluminium walls	T-PEPT	5	0.64 (15)	0.97 (15)	0.67 (15)
	PEPT-ML	5	0.83 (14)	1.16 (14)	0.91 (14)
	BHAM Method	4	1.2 (3)	2.0 (3)	1.4 (3)
Glass tracer, steel walls	T-PEPT	5	1.84 (19)	2.28 (19)	1.94 (19)
	PEPT-ML	5	2.25 (19)	2.68 (19)	2.31 (19)
	BHAM Method	4	3.8 (4)	4.6 (4)	3.8 (4)
Steel tracer, steel walls	T-PEPT	5	2.56 (14)	2.90 (14)	2.66 (14)
	PEPT-ML	4	4.2 (4)	5.1 (4)	4.3 (4)
	BHAM Method	3	7.4 (8)	9.2 (8)	7.9 (8)

## Discussion

The results from Fig. 5 show that T-PEPT successfully identified all tracers, even with the largest number: 79. Further, T-PEPT consistently had one of, if not the, lowest errors for both the ADAC and ECAT detectors, indicating its robust performance and ability to handle a large number of tracers in random positions. The error increases slightly in the ADAC with the number of tracers whilst remaining relatively constant across the number of tracers in the ECAT detector. This error plateau suggests the algorithm has a high degree of scalability, comparable with the PEPT-EM algorithm and in particular PEPT-ML. Analysis of all other benchmarking tests and how T-PEPT performed can be found in the Supplementary Material Section 3.

From Fig. 6 it is clear that all algorithms struggle to find a smooth trajectory in the steel tracer-steel walls scenario due a high proportion of false to true tracer locations. However, the errors on T-PEPT are smaller than both PEPT-ML and the Birmingham Method, making T-PEPT’s ability to still track all five tracers more impressive. Indeed, across all mill scenarios, T-PEPT’s average spatial errors in X, Y, and Z were smaller than both PEPT-ML and the Birmingham Method. Further, from Table 1 we see that T-PEPT was able to track all five tracers simultaneously in all scenarios, including the highest-noise environment, whereas PEPT-ML and the Birmingham Method only detected four or three tracers for the full 10 rotations.

The high attenuation of steel tracers, steel ball surroundings and steel walls meant that these other two algorithms were unable to reconstruct evert tracer location for the full duration. Unlike conventional methods that rely on optimising specific trajectory fits, TDA identifies and tracks persistent topological features within noisy datasets. This approach reduces the impact of false LORs and scattered coincidence events, allowing T-PEPT to consistently identify tracer paths even in environments with significant noise. In the steel tracer with steel walls scenario, T-PEPT’s ability to prioritise persistent structures ensured complete trajectory reconstruction where other methods were less effective.

T-PEPT consistently exhibits the lowest STD values across all scenarios, signifying a higher consistency and stability in T-PEPT’s accuracy for locating the true tracers’ positions. In other words, T-PEPT not only achieves accurate localisation but also maintains uniform performance across different measurements, even in high-noise environments. This contrasts with the higher STD values observed for PEPT-ML and the Birmingham Method, which suggest greater variability and less reliable trajectory reconstruction under the same conditions.

In the high-noise scenario, the Birmingham Method’s tracking capabilities clustered multiple tracer trajectories together, only locating three tracers instead of all 5. This result is most likely due to how the Birmingham Method tracks multiple particles - the tracer with the highest data logging rate is chosen to be the first particle, then so on as the algorithm iterates^34,35^. However, in this case, all the tracers have exactly the same activity, so the algorithm struggles to decide which cluster belongs to which tracer, thereby assigning some points to the wrong cluster. T-PEPT and PEPT-ML both have the capacity for ‘two-pass’ clustering^8^, thereby allowing for greater refinement of the location of the cluster centres. However, PEPT-ML also seemed to struggle to separate tracers close together, thus only locating four our of the five tracers in the most challenging scenario, and also had higher positional errors than T-PEPT in all cases, with some being more than double.

## Conclusion

T-PEPT has demonstrated itself as a novel and effective algorithm for tracking multiple tracers in high-noise environments. While the Birmingham Method and PEPT-ML have their respective strengths and have been foundational in the development of PEPT techniques, T-PEPT offers a complementary approach that performs extremely well in scenarios involving complex, high-noise datasets. By incorporating topological data analysis, T-PEPT is able to capture the underlying structure of particle trajectories, filtering out noise and identifying persistent features that correspond to true particle paths. This topological insight enhances the algorithm’s robustness and adaptability, enabling it to maintain low positional errors and successfully identify all tracers within difficult conditions. The algorithm’s scalability and precision, combined with the advantages of topological methods, make it a valuable addition to the toolkit for radioactive particle tracking, particularly in challenging experimental settings.

## Supplementary Information

Supplementary Information.

## Data Availability

Data for the PEPT standard testing framework can be found here https://github.com/mxh1092/RoPP-Comparison-Functions.
