# Supplementary material for: A topological approach to positron emission particle tracking for finding multiple particles in high noise environments
Source: Sci Rep. 2025 Apr 19;15:13599. doi: 10.1038/s41598-025-97175-0 (PMC12009289; doi:10.1038/s41598-025-97175-0)
Supplement: Supplementary file 1 — Supplementary Information. [file 41598_2025_97175_MOESM1_ESM.pdf]

Supplementary Material

Manuscript Title

A Topological Approach to Positron Emission Particle Tracking for Finding Multiple Particles in High Noise Environments

Authors

Jack Alan Sykes, Andrei Leonard Nicuşan, Dominik Werner, Matthew Herald, Daniel Weston, Tzany Kokalova Wheldon, Christopher Windows-Yule

1 Topology

Topology is a branch of mathematics that studies the continuity and connectivity of intrinsic geometric properties of higher-dimensional surfaces and manifolds within a space including the shape, the number of holes and connected components. In other words, topology is the study of qualitative geometric information [1, 2, 3]. The discipline dates as far back to Euler when solving the famous Königsberg bridge problem in 1736 [4], and Gauss studying the linking number of the orbits of asteroids in the 19th Century [5]. The Königsberg bridge problem, solved by Euler, involved determining whether it was possible to cross every bridge in the city once without repeating a crossing [4]. This problem laid the groundwork for graph theory, a branch of combinatorial topology. Similarly, Gauss's study of asteroid orbits introduced the concept of linking numbers, foundational to knot theory [5].

The field has significantly advanced over the years, covering an extensive array of topics such as knot and graph theory, topological optimisation, topological data analysis and topological materials and devices, to name a few broad applications [2, 3, 6]. Modern applications of topology span numerous fields. In computer science, topology aids in data analysis and network theory such as analysing the spread of epidemics [7]. In physics, it contributes to understanding the properties of exotic states of matter [8], while in biology, it assists in studying the shapes and folding patterns of molecules [9].

In the field of topology, the intrinsic geometric properties are invariant under continuous deformation: objects that are twisted and stretched as opposed to cut and glued together [1]. For example, a doughnut and a coffee cup have the same topology, as one can be transformed into the other through continuous deformation without cutting or attaching extra components. Additionally, the study of topology is in a space such that it is irrespective of any measure of length or distance function (also known as a *metric* [10]), i.e. there is no coordinate system-dependence. Distance functions are instead replaced with the concept of infinite nearness of a point to a set of neighbourhoods, or a subset, of the underlying space. This disregard for the metric allows for the qualitative study of datasets as opposed to metric-dependent geometric analysis, which is inherently more quantitative [2, 3].

Topology is often divided into four main sub-fields: General or Point Set Theory, Combinatorial Topology, Differential Topology, and Algebraic Topology - the last being the main branch used in this work. Algebraic topology, in particular, examines how algebraic structures can describe topological spaces. Concepts like homotopy and homology are fundamental in understanding how shapes can be continuously transformed into one another and classifying spaces by their holes, respectively. Homotopy describes when one shape can be continuously transformed into another without cutting or gluing, while homology is a mathematical tool used to classify topological spaces based on the number and types of holes they have at different dimensions [2, 3].

This paper does not aim to give an in-depth account of topology and all its contents and applications: it will instead give an overview of the relevant tools that algebraic topology possesses that are key to the understanding and development of a proposed topological-based PEPT algorithm. The *T-PEPT* algorithm leverages these principles, utilising tools from topological data analysis, notably persistence homology, to capture the multi-scale structure of data crucial for understanding complex particle trajectories in positron emission particle tracking.

For more detailed and mathematical descriptions of topology, please see work done by Carlsson [2], Kelly [3], and Kuratowski [11].

1.1 Algebraic Topology

Algebraic topology is a significant branch of mathematics that intertwines the concepts of algebra and topology. It focuses on the global properties of spaces, employing algebraic structures such as rings, groups and high-dimensional structures to study topological problems. This field has developed various methods and concepts to analyse the shapes and structures of topological spaces within an algebraic framework [2, 12, 13].

Algebraic topology applies to *point clouds*: finite sets of points with a distance function in space. These point clouds serve as discrete approximations of geometric objects and can be gathered from real-world data, including 3D scanning and motion-capture data. Therefore, algebraic topology is a suitable approach to PEPT trajectory reconstruction using points clouds to represent the PEPT data. As described in the PEPT Methods Section of the main text, LORs can be converted into points, or point clouds, via calculating the 'cutpoints' of the LORs. The field provides tools to analyse these point clouds by constructing *simplicial complexes* (see Section 1.2.1). These complexes are geometric representations of topological spaces, built from points, line segments, triangles, and their higher-dimensional analogues. This approach is invaluable in identifying and recognising global features from data samples, even in the presence of noise [2, 14].

At the core of algebraic topology are homology and cohomology groups. These groups classify topological

spaces based on their structure. For instance, homology groups (see Section 1.3) distinguish between different shapes, such as a torus and a Klein bottle, by assigning algebraic structures that reflect their number of holes and voids. Fig. 1 depicts a torus and a bottle. The distinction is based on the different homology group values these shapes possess. Conversely, cohomology groups offer a dual perspective, focusing on the functions defined on the spaces rather than the spaces themselves [2, 12, 13]. Cohomology will not be a focus of this paper - the interested reader can find more on cohomology here [15, 16].

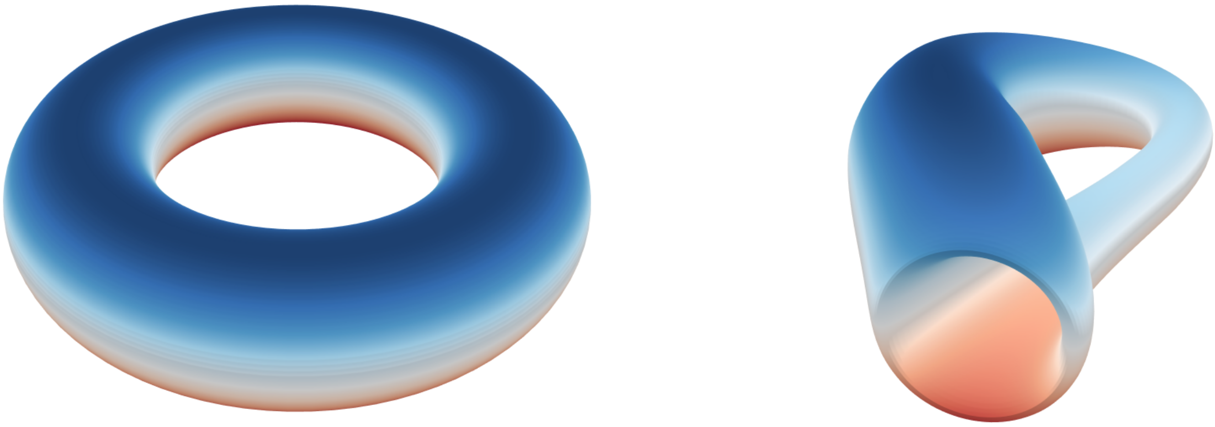

Figure 1: (Left) A torus, a doughnut-shaped surface with a single hole, representing a classic three-dimensional object. (Right) A Klein bottle, a surface that loops back into itself, encapsulating a four-dimensional paradox in three-dimensional space. The Klein bottle is a single surface and thus does not have a hole.

Homotopy groups are another fundamental concept in algebraic topology, studying the properties of spaces preserved under continuous deformations, such as stretching or bending, but not tearing or glueing. Homotopy groups are adept at capturing the essence of 'shape' in a topological space and classifying them, providing insights into its connectivity and recording the information on holes of different dimensions. If two mappings can be continuously deformed into one another they are said to be homotopic [13, 17]. A simple example of where two objects are not homotopic is the torus and the sphere: the torus has a hole and the sphere does not, therefore they are neither homotopic nor topologically equivalent.

The techniques developed in algebraic topology find applications in diverse fields, from data analysis to theoretical physics. In data science, Topological Data Analysis (see Section 1.2) employs methods from algebraic topology to extract meaningful patterns and shapes from complex data. In theoretical physics, the field aids in understanding the properties of exotic spaces and fields. Algebraic topology offers a unique and powerful set of tools for understanding and analysing the shapes and structures inherent in topological spaces. Translating topological problems into algebraic terms opens up new avenues for addressing complex problems across various scientific disciplines [2, 12, 13].

1.2 Topological Data Analysis

Frequently, data is represented as a series of points in Euclidean and Real n-dimensional space, $\mathbb{E}^n$ and $\mathbb{R}^n$, respectively, with little or no ordering. Topological Data Analysis (TDA) addresses problems that data analysis commonly encounters. Specifically, TDA addresses how high-dimensional data structures can be represented in low-dimensions, and how discrete data points can be constructed into a global structure [18, 14]. This representation is achieved by restructuring the dataset into a group of *simplicial complexes* (see Section 1.2.1) and applying *persistent homology* to the set - a form of algebraic topology (see Section 1.3) [18, 14].

Topological Data Analysis represents a significant breakthrough in understanding complex data structures by leveraging concepts from topology and algebra and is particularly effective for point cloud data (see Section 1.1). TDA can be viewed as a continuation of the mathematical tradition of organising infinite sets into coherent structures. It employs metric spaces, equipped with a distance function satisfying specific axioms, to understand and study shapes. This methodology is not limited to conventional two or three-dimensional shapes but extends to higher-dimensional analogues and more abstract geometric forms. In TDA, the notion of a metric space becomes an essential tool for organising large but finite data sets [2, 18].

TDA is very robust to noise due to it studying the shape and patterns within data rather than concerning the distance between points and other geometric properties. In high-dimensional spaces, where the "curse of dimensionality" makes traditional analysis challenging, TDA's ability to focus on global structures rather than

individual dimensions gives it a significant advantage. TDA also possesses multi-scale analysis capabilities, identifying key features of a dataset in different dimensions. High-dimensional data tends to be sparse and has more relevant low-dimensional features, which other clustering algorithms may struggle with [2, 18, 19].

Topological Data Analysis, through its unique approach to organising and understanding data, offers a profound way to extract meaningful information from complex data sets. It bridges the gap between abstract mathematical concepts and practical data analysis, providing a robust framework for uncovering the hidden structures within data.

1.2.1 Simplicial Complex

Formally, a p-simplex is defined as the convex hull (the tightest enclosure around a set of points such that any line segment drawn between two points in the enclosure never leaves the set) of $p + 1$ linearly independent points in Euclidean space and is therefore determined by its $p + 1$ vertices. The convex hull can be likened to stretching a tight elastic sheet over a set of points, forming the smallest enclosing shape analogous to the p-simplex. A simplicial complex is formed from the combination of 0-, 1-, 2-, 3-simplices and so on in higher dimensions, corresponding to vertexes, lines, triangles, tetrahedrons, etc., respectively [2, 18, 20]. The simplicial complex can be constructed using any possible combination of these simplices, with each simplex not having to be connected to every other complex - as can be seen in the right-hand graph of Fig. 3(a) in the main text.

Simplicial complexes can be formed through the concept of "closeness" of points, whether this be Euclidean distance or any other metric dependent on the application. If two points are considered to be 'close' an edge (1-simplex) is connected between two 0-simplices (vertices). One way a simplicial complex may be constructed is through the 'growing' of circles around points in two-dimensional Euclidean space, $\mathbb{E}^2$. As the radius of the circle increases, the circle intersects with the circles corresponding to other data points; when this happens, an edge is connected between the two points, as they merge to form a new 'connected component'. This can be seen in Fig. 3(b). When multiple edges combine they start to form triangles, tetrahedra and so on in higher dimensions, taking note of the resulting loops and holes/voids in the data also. The edge merging continues until eventually a complete set is constructed in which every data point is connected via an edge with every other data point, or some pre-defined threshold has been met. The formation of simplicial complexes is key to understanding the underlying 'shape' of the data and how topological features and structures can arise from them [2, 21].

In Fig. 3(b), we illustrate two types of simplicial complexes in the $\mathbb{R}^2$ plane: the Čech and Vietoris-Rips (V-R) complex. Despite their apparent similarity, they differ in their approach to forming loops, triangles, and higher-dimensional shapes by connecting vertices. In the left figure, the Čech complex forms two triangles (coloured lilac), or closed loops, by requiring that all vertices within a shape must share a common intersection point; thus, among the three potential loops, only two form triangles due to their vertices all intersecting. On the other hand, the V-R complex, as shown on the right, constructs higher-dimensional shapes by simply connecting vertices pairwise. This method results in one triangle and a tetrahedron; the tetrahedron emerges because the vertices of the lower triangle and the single vertex above are pairwise connected, fulfilling the condition for a tetrahedron: a 3-dimensional shape with four triangular faces. Consequently, the dimension of the Čech complex is identified as 2, as it fits within the $\mathbb{R}^2$ plane, whereas the dimension of the V-R complex is considered to be 3, indicating its structures cannot be fully represented, or embedded, in $\mathbb{R}^2$ [18]. To summarise, if all 3 points' radii are connected pairwise but do not overlap at a single point then the simplicial complex is V-R and not Čech.

The choice of the simplicial complex depends on the application: using the Čech complex in three or higher-dimensional space can quickly become computationally expensive, as the algorithm would need to check that every circle, sphere or similar metric for 'closeness' is intersecting for a particular shape to be formed, whereas the V-R only considers if its neighbours are connected to its other neighbours [22]. There are various pros and cons for using either complex, and others exist, but for the computational benefits, the V-R complex was chosen in the development of T-PEPT.

By replacing a dataset of points with a family of simplicial complexes, the dataset is now in the form of global topological objects that can be analysed on different scale lengths [14].

1.3 Persistent Homology

1.3.1 Homology

Homology is a mathematical tool to provide an algebraic description of topological spaces. Mathematically, we let X be a space containing a family of abelian groups $H_0(X)$, $H_1(X)$, ..., $H_n(X)$, ..., each assigned to a continuous map $f\colon X \longrightarrow Y$ where Y is a family of group homomorphisms $f_n\colon H_n(X) \longrightarrow H_n(Y)$ [23].

An *abelian* group is one in which the group operation is commutative, i.e. when applying an operation to the elements of the group the order in which they are applied is irrelevant; in other words: $A\ B = B\ A$ for all elements A and B [23].

A *homomorphism* is a map between two algebraic objects of the same type (i.e. two groups or two vector spaces) which preserves the structure of the algebra and its operations. Applied to the mapping above, $f\colon X \longrightarrow Y$, and if $\cdot$ represents an operation of the structure, then $f(x \cdot y) = f(x) \cdot f(y)$ for every pair elements x, y in the binary example case [23].

More simply put, spaces are analysed in terms of their homology groups $\mathrm{H}_n(\mathrm{X})$ where each group $\mathrm{H}_n(\mathrm{X})$ is associated with a distinct dimension n. These groups are constructed from chains of n-dimensional simplices that map the underlying structure of the space X, offering a way to understand their properties algebraically rather than solely through visual or geometric means. At its core, homology is concerned with the identification and classification of n-dimensional holes within a topological space. Here, a "hole" is defined in a generalised sense, extending beyond simple gaps or voids to include any topologically significant feature that contributes to the space's overall structure [23, 20, 24]. Specifically:

- The Zeroth homology group (H_0) concerns the connected components of a space. Each component corresponds to a set of points that are connected in some manner, allowing us to quantify the space's degree of connectivity or isolation. This can be either individual vertices or vertices connected by edges.

- The First homology group (H_1) captures loops or cycles in a space that do not bound any fillable surface. These non-bounding loops, visualised in Fig. 2, are the 'holes' of the space — unfillable gaps that remain even if the shape is stretched or squashed, hinting at the space's intrinsic cyclical structure. In 2D, this is like a line drawn around an empty space without enclosing any surface, and in 3D, it is like a string looping through a doughnut or around its entirety without being able to lay flat against it.

- The Second homology group (H_2) and higher (H_n for $n > 2$) generalise this concept further to identify holes of higher dimensions. For instance, H_2 detects voids or cavities that are enclosed by surfaces but not filled in, akin to the hollow inside a sphere.

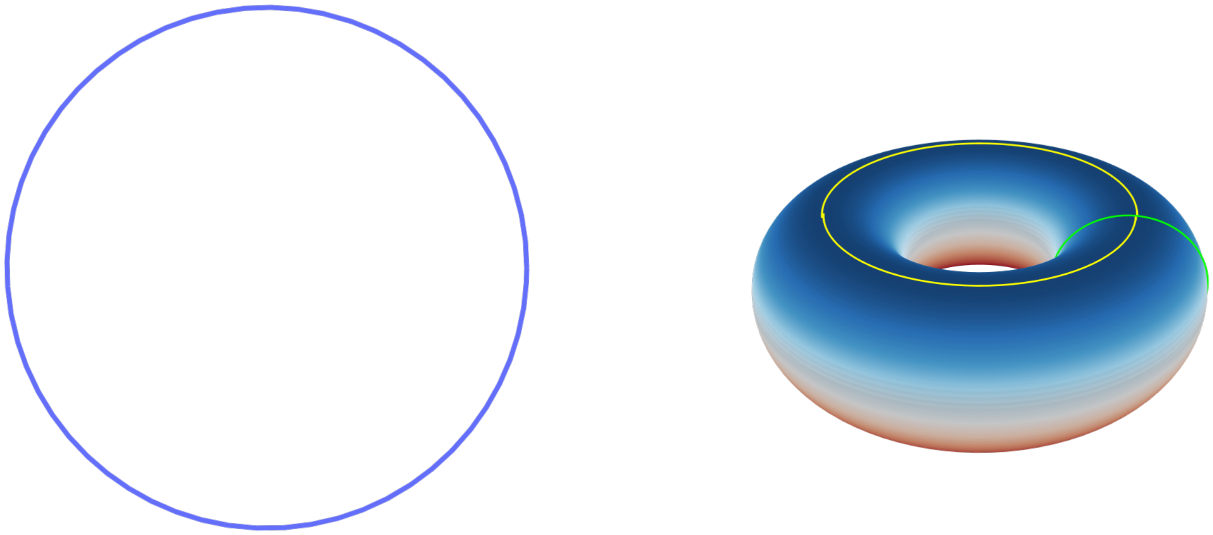

Figure 2: Figure illustrating 'holes' in relation to H_1. On the left, a circle represents a 2D 'hole' that a 1D boundary cannot fill, forming a gap in the plane. On the right, the torus displays 3D 'holes': the yellow loop outlines a central cavity while the green loop runs along the torus' body. Neither loop can be seamlessly covered by a single 2D sheet, showcasing the 3D 'holes' that remain open in the fabric of the space, as defined by H_1.

By translating the complex and often intangible characteristics of topological spaces into a structured algebraic framework, homology provides a powerful means to analyse and compare spaces in a manner that is

both rigorous and insightful. It bridges the gap between abstract topological intuition and concrete algebraic analysis, enabling mathematicians and scientists to uncover the underlying structure of spaces that are otherwise difficult to visualise or comprehend in purely geometric terms [23, 20, 24].

1.3.2 Persistence

To compute how topological features reveal information about a dataset, analysis of the underlying shape of the dataset is required using *persistent homology.* Persistent homology computes the topological properties of the dataset and studies them over a large range of spatial resolutions, e.g. the 'growing of circles' previously mentioned, albeit spheres in this case. Robust features will be found at a large interval within this range, whereby they are known to '*persist*' and represent the true nature of the underlying shape of the dataset. Other, less persistent features will be found in smaller range intervals and are deemed less important, or simply noise [21, 20]. To evaluate the importance of topological properties, a *filtration*, or topological 'simplification', is required. Simply put, the filtration ranks, and subsequently removes topological attributes in the order of their increasing importance, with the removed attributes being assigned as noise [21]. The filtration is chosen based on the application, for example, a density-based filtration may be used for points in 3-dimensional space.

Persistent homology is applied to a dataset represented as a simplicial complex, as discussed in Section 1.2.1, with the filtration being applied to the complex. A filtration, or rather a 'filtration complex', can be described as a series of simplicial complexes increasing in both size and complexity. These filtrations are also represented as a nested sequence of increasing subsets [18, 20]. The filtration can be stopped by the users at any time, but conversely, it can continue until a complete graph has formed: a graph is complete if every two 0-simplices (vertices) are connected by a 1-simplex (an edge) [25].

The persistence homology of a dataset is most commonly displayed as a 'Birth-Death' persistence diagram, as shown in Fig. 4(b) in the main text. Here, the homology groups are displayed in different colours, which represent the connected components (H_0), the loops/cycles (H_1) and the holes/voids (H_2) as outlined in Section 1.3.1. The purpose of the birth-death plot is to show how topological features change or emerge over a scale of filtrations - in this example, the filtration corresponds to the 'maximum edge length', i.e. the maximum Euclidean radius of the spheres formed around each point using a Vietoris-Rips simplicial complex. Essentially, it is the Euclidean distance at which point the spheres surrounding each point intersect. If the filtration value was too low, say less than 0.5, we would not see the formation of the persistent hole inside the hollow sphere - the very thing that defines its structure. If the filtration value is too high, this will become computationally expensive as each point will have to identify connections with more neighbouring points.

All connected components (which start as simply vertices) are born at zero, where zero simply means that the radius of the Euclidean sphere (in this example) growing around each point is zero. As the filtration increases, some vertices merge via edges, and one vertex is said to have 'died', leaving the other to continue. It is at this "death" that the point is plotted on the diagram; in the case of the hollow sphere point cloud in Fig. 4 in the main text, all connected components were formed and died within filtration value of just over 0.2. Many loops within the sphere were formed as the filtration value increased, with some dying immediately and some taking longer to die; the latter is known as being persistent. In particular, this persistence is evident in the second homology group, whereby a single hole or void persists far greater than any other hole in the dataset. This makes sense, as we know with a hollow sphere there is a large hole inside of it. However, if we did not know this information *a priori*, this is the type of information that TDA can give us about the shape of the data.

Persistence homology can reveal the most important, or persistent, pieces of information within a dataset regarding its underlying shape and structure. This process can be applied to a multitude of data types, however, and does not have to be a 'physical' shape such as a point cloud hollow sphere. Persistence homology has been applied to Geospatial data contained within spreadsheets [26], neural networks [27] and even quantum entanglement data [28].

1.4 Topological Mode Analysis Tool (ToMATo)

The Topological Mode Analysis Tool (ToMATo) is a powerful method for identifying the intrinsic structure of complex datasets by analysing the shape and connectivity of data to identify patterns and relationships in data. ToMATo is regularly used as a clustering algorithm, therefore elements of this algorithm have inspired the development of T-PEPT. A brief step-by-step guide to ToMATo is described below and the relevance of each point to PEPT is outlined. For more information please read the work by Chazal *et al.* [29].

ToMATo combines abstract mathematical concepts with practical data analysis applications based on the persistence of topological features, providing an innovative and informative perspective on data analysis [29]. ToMATo has been applied to and influenced a diverse range of fields, demonstrating its effectiveness and

versatility in analysing complex datasets. Recently, ToMATo has been applied to biomedical research such as cell segmentation in fluorescence microscopy image analysis [9, 30].

From a clustering point of view, the principles of ToMATo are:

1. Construct a simplicial complex on the dataset, such as the Vietoris-Rips (V-R) complex (see Section 1.2.1), serving as a foundational structure for capturing the relationships and shapes within the data from a topological perspective.
   - PEPT data can be converted into point cloud data and the V-R complex is constructed on the points, building up a network to understand the relationship between each point.
2. Starting with an empty complex tree, add filtrations (a nested sequence of simplices) by order of their 'importance' - typically in ToMATo, these filtrations may be based on the density of the points or their distance to their neighbours or some other metric that emphasises the most significant topological features first.
   - Tracer clusters in PEPT tend to have high regions of density whereas areas of noise tend to be sparser, therefore filtrations can be used to identify these dense regions and order their importance, i.e. potential tracer-related points before noise-related points.
3. Apply persistence homology to the data as the filtration progresses to measure the 'lifetime' of the topological features across the homology groups. This analysis helps to distinguish between short-lived topological noise and more persistent, meaningful structures.
   - Identifying which points, or cluster of points, persist the longest gives an indication as to which points will belong to the tracer(s).
4. Plot the birth-death persistence diagram and identify persisting topological structures in the data. From observing the plot, set a 'threshold' to filter out transient features and consider them as noise allowing to focus the analysis on significant topological characteristics.
   - Tracer clusters should stand out amongst the noise in PEPT data, therefore thresholding to consider these points only could reveal the number of tracers in the dataset.
5. Re-run the algorithm with the newly established threshold value, assigning these persistent features as the core clusters of the dataset data. ToMATo outputs the clusters as sets of data points grouped based on their shared topological significance [18].
   - This step is not as applicable to PEPT as however it can act as a check on the chosen parameters, further fine-tuning the clustering algorithm.

Implementing ToMATo from existing libraries was slow and memory intensive, therefore only the relevant steps for T-PEPT were utilised, specifically steps 1-3 and step 4 being an optional step for the user - see Section 2 for more details.

ToMATo is an iterative approach that relies on user input of a parameter, *tau*: the threshold value used to create clusters based on persistence values. When traversing the filtration, if two clusters are within a 'distance' (depending on the filtration metric chosen) less than or equal to tau, they are merged into a larger cluster. This merging is reflected on the persistence diagram as a point, signifying the death of one cluster as it combines into another. This helps the avoidance of multiple small clusters and focuses on larger, more topologically persistent, clusters. Clusters with, say a maximum difference in density (filtration value) larger than tau, will remain as separate clusters [29].

After the points are assigned filtration values, they are sorted within a simplex tree: in the example case of density being the filtration metric, the densest values are sorted first. Iterating through these sorted values, the points are assigned to a cluster, with their filtration value being tracked. When adding a new point to a cluster, the filtration value of the new point is compared with the maximum value of the cluster; if the difference between filtration values is larger than tau, a new cluster is created. Otherwise, the point is added to the existing cluster and the maximum filtration value is updated if necessary [29].

Fig. 3 demonstrates how a threshold value might be applied to a real dataset example. Consider the hollow sphere point cloud from Fig. 4 in the main text in Section 1.3.2. As stated previously, the filtration is the 'maximum edge length', i.e. the Euclidean radius of the spheres formed around each point using a Vietoris-Rips simplicial complex. If a threshold is set to, say, a value of 0.4, we can see that there is only one point that lies above the threshold line, corresponding to a single void in the H_2 group, equal to a hole in the hollow sphere.

Using this threshold value, the ToMATo algorithm is run again, and the output would be a single cluster in the second homology group, and no clusters in the zeroth and first homology groups. Of course, this example is a very specific case, and using datasets without *a priori* information will give differing results across all homology groups.

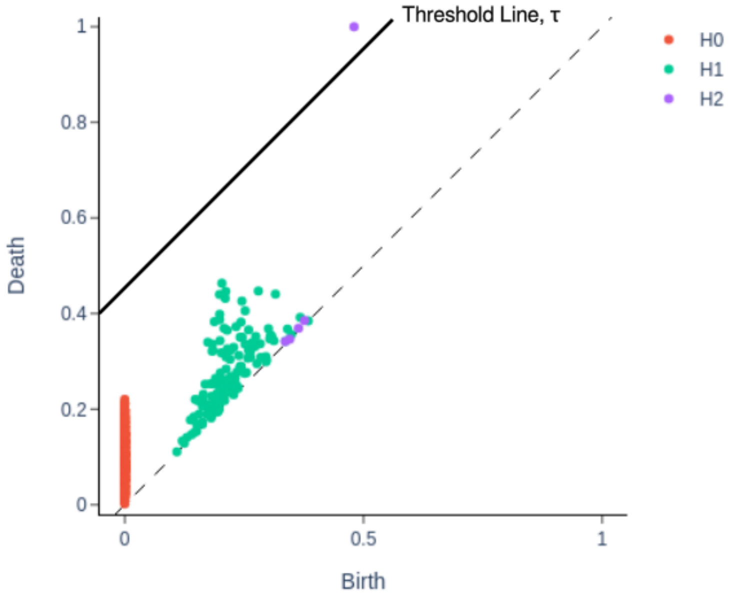

Figure 3: The persistence diagram of the hollow sphere from Fig. 4 in the main text showing homology groups H_0, H_1 and H_2 as well as a threshold line with value $\tau = 0.4$

2 The Algorithm: T-PEPT

2.1 Data Segmentation and Cutpoints Calculation

PEPT data is output in the format of time-consecutive LORs as (t, x, y, z) (as well as any other extra information such as trajectory label) corresponding to the time and spatial positions that a tracer was detected at each detector. For example, for the Philips ADAC Forte dual-headed positron camera, the data is output as (t1, x1, y1, z1, t2, x2, y2, z2) corresponding to the two detector heads [31]. Thousands of LORs are recorded every second by the PEPT detectors, therefore, to keep track of the tracer movement over time the LORs are split into smaller samples that can be processed in turn. Each sample is used to compute one pseudoinstantaneous position per tracer present. The sample size is a free parameter that needs to be carefully chosen: large enough to accurately determine the tracer position, but small enough that it can accurately track the tracer without significant movement. Due to these constraints, a standard sample size ranges between 100 and 250, however, this value depends on the dataset in question. Further, a smaller sample size improves the temporal resolution of the PEPT data [32, 33, 34, 35].

T-PEPT utilises the PEPT library to transform PEPT data into the correct readable format. The data is first converted in LORs using the `pept.LineData()` method and, using a suitable parameter value for the 'sample_size', the 'cutpoints' are computed and initialised as a subclass of the `PointData` class. The cutpoints are defined as the point that minimises the distance between every pair of LORs and each sample of said cutpoints is clustered using HDBSCAN (Section 2.5.1). For more detail on specific PEPT library classes and further details on cutpoints see 'Positron emission particle tracking using machine learning' by Nicuşan *et al.* [35] and the University of Birmingham Positron Imaging Centre PEPT GitHub page here.

2.2 Topological Clustering

Based on the principles and first few steps from the ToMATo clustering algorithm [29] (see Section 1.4, a Gaussian Kernel Density Estimation (KDE) is applied to the cutpoints in each sample to identify regions of high density within the dataset. A k-dimensional (k-d) tree is then created to query the distances of the points' nearest neighbours. K-d trees are structures that partition and organise points within k-dimensional space and are widely used in nearest neighbour searches and constructing point clouds [36]. In the context of T-PEPT, the k-d tree is used to find the nearest neighbours for each point within the space, relating the density value of each point to its spatial context by effectively identifying its surrounding points. By checking only the nearest neighbours, the need to check every point against every other point is void, thus reducing complexity and speeding up the process of identifying connections between points.

A simplex tree, a data structure for analysing topological data, is used to manipulate and store simplicial complices. The simplex tree is particularly useful in persistent homology computations as it manages the construction of simplices through the filtration process, keeping track of all faces within the simplicial complex, and therefore the vertices, edges and their interconnections, in a hierarchical manner [37]. The simplex tree is implemented using the GUDHI open-source C++ library with Python interface (see here).

The index of each point in the sample is inserted into the simplex tree with a filtration value equal to its density, as determined by the Gaussian KDE. This gives each vertex a weight in the simplicial complex, representing a local density of the data surrounding that point. The k-d tree is queried to find the nearest neighbours for each point, determining which points are close enough to each vertex to form an edge between them and build up the simplicial complex. The inserted edges are assigned a filtration value also, determined by the average density of the two points, giving it a weight. As each point and edge is added to the simplex tree their indices are saved in an array, keeping track of their original position in the dataset.

To explain this further, in contrast to simple filtration — where the construction of simplicial complexes is driven by the straightforward expansion of geometric shapes like growing spheres around data points, capturing connections based on proximity — the filtration technique employed in T-PEPT leverages Gaussian Kernel Density Estimation. This approach does not merely consider the Euclidean closeness of points: instead, it assesses the density of data points, akin to recognising areas of intense gravity in a celestial map. As such, the simplicial complex becomes weighted, not just by the mere distance between points, but by the significance of their density. This density-centric filtration allows for a more detailed interpretation of the dataset's shape, revealing topological features that hold meaning not only in the context of spatial nearness but in the underlying architecture of the data distribution. The k-d tree aids in this refined process by efficiently pinpointing the nearest neighbours within this context of density, rather than across the entire dataset, thereby streamlining the construction of a simplicial complex that truly represents the topological differences of the data. To clarify, this clustering step is based solely on topological information and not spatial information.

In the simple Euclidean distance case where the filtration is the increasing radius, r, of a ball around each point, p, we denote the Euclidean filtration as:

$$f_E(p) = r \tag{1}$$

Here, the ball grows uniformly for all points as r increases. In the Gaussian KDE-weighted case the filtrations consider the density at each point, p, and for each edge, e:

$$f(p) = \rho(p) \tag{2}$$

$$f(e) = \frac{\rho(p_1) + \rho(p_2)}{2} \tag{3}$$

Where p_1 and p_2 are two different points that the edge connects. The overall filtration process, $\mathcal{F}$, can be described as a collection of sub-level sets of the density function ρ, where each sub-level set $\mathcal{F}_a$ for a given filtration level a includes all points and edges with a filtration value $\leq a$:

$$F_a = \{p \in X | f(p) \leq a\} \cup \{e = (p_1, p_2) \in X \times X | f(e) \leq a\} \tag{4}$$

Below is a breakdown of equation 4:

- $\mathcal{F}_a$ is a collection of all points and edges that have a filtration value $\leq a$. As a increases, more points and edges are included; inclusion is based on whether their density *f(p)* (for points) or average density *f(e)* (for edges) is $\leq$ the current value of a.

- $\{p \in \mathrm{X} — f(p) \leq a\}$ - this part includes a point p in the set $\mathcal{F}_a$ if the density of p (filtration value $f(p)$) $\leq a$.
- $\{e = (p_1, q_2) \in \mathrm{X} \times \mathrm{X} — f(e) \leq a\}$ - this part includes an edge e connecting points p_1, p_2 in the set $\mathcal{F}_a$ if the average density of p_1 and p_2 (filtration value $f(e)$) $\leq a$. X $\in$ X denotes the Cartesian product of the dataset with itself, representing all possible pairs of points (p_1, q_2), thus considering every potential edge e within the space for inclusion based on the density criteria.
- $\cup$ is the union, which combines the set of points with the set of edges that meet the criteria.

The complexity of the filtration varies depending on the chosen metric, particularly compared to the simple Euclidean filtration seen in equation 1.

Once the simplex is constructed, the filtration values for all points and edges are extracted and passed through a 1D Hierarchical Density-Based Spatial Clustering of Applications with Noise (HDBSCAN) clustering algorithm [38, 39]. HDBSCAN converts the Density-Based Spatial Clustering of Applications with Noise (DBSCAN) into a hierarchical clustering algorithm. The space is first transformed according to the density of the points, where denser areas are brought closer together, and sparser areas are moved further apart. A minimum spanning tree is created to represent the hierarchical density-based clustering, where clusters are identified as the branches of the tree that persist over a wide range of scales, implying a stable cluster. This is known as the single-linkage method. In particular, HDBSCAN is fairly robust to noise and adept at identifying clusters of differing densities, where it returns the number of clusters and data points corresponding to them. HDBSCAN has a high-performance implementation, ideal for larger datasets. For more information on DBSCAN and HDBSCAN, please read Campello *et al.* [38] and McInnes *et al.* [39], and for more detail on how HDBSCAN is implemented into the PEPT library please read Nicuşan *et al.*[35].

It is worth remembering that this process is not clustering the points themselves to identify tracer clusters, the clusters within the filtration values are more abstract and relate to the density and connectivity within the data across scales, rather than physical locations or entities. Clusters within filtration values signify groups of data points that become significant around the same scale. These clusters reflect the emergence of topological features as the filtration parameter is varied.

2.3 Noise-to-Signal Ratio Calculation

When clustering filtrations, we are looking for persistent features across scales—these are the real 'signals' in the data amidst the 'noise'. Once clustered into groups via HDBSCAN, the filtrations are assigned labels referring to their cluster group number (0, 1, 2, ...) or whether they are grouped as noise (-1). This process is applied to both the points and the edges, yielding signal and noise clusters for each. Deciding whether an edge is noise or signal is determined by its vertex constituents - if the tuple of vertices associated with the edge are both labelled as noise points, then the edge is also labelled as noise. The number of points and number of edges are summed for signal and noise to inform how much of the data contributes to meaningful structures versus outliers or irrelevant structures.

Traditionally, the true_fraction parameter is defined as the ratio of signal-to-noise, as is used in the PEPT-ML algorithm [40]. However, in the approach described in this paper, the true_fraction parameter has been redefined to directly quantify the level of noise within the dataset, known as the noise-to-signal ratio. This change in definition is the result of empirical observation that the measure of noise within the topological structures within a variety of types of datasets (see Section 3) yielded better clustering outcomes than measuring the signal directly. By focusing on the noise level within the dataset, T-PEPT effectively adapts to the unique characteristics of each dataset, ensuring that the clustering results are both accurate and meaningful.

In the context of T-PEPT, a low true_fraction value indicates a large proportion of noise in the filtrations meaning that most of the data does not contain stable, dense clusters. Conversely, if the true_fraction value is high, there is little noise and coherent structures are more apparent. The calculation of the true_fraction alleviates the need for manual parameter adjustment, automating the processing pipeline further by allowing the algorithm to adaptively refine its clustering methodology. The true_fraction value is used to define parameters in a two-pass-clustering stage using HDBSCAN (see Section 2.5.1).

2.4 (Optional) Plotting a Persistence Diagram

Within T-PEPT the user can call the method 'calculate_persistence' where persistence homology is directly applied to the dataset to produce a persistence diagram. The function begins by calculating the 'max_edge_length' by considering the maximum dimension-wise spread of the data, specifically between the 10th and 90th quartile

to exclude extremities affecting the scale. The max_edge_length is equal to the maximal difference between the two quartiles multiplied by a constant, k, where $k \in \{1, 2, 5, 10, 20, 50, 100\}$. This multiplication, as seen in Equation 5, is to ensure that a persistence diagram is computed at the correct scale where the topological features are most meaningfully represented.

$$max\_edge\_length = k * max(q_2 - q_1) \tag{5}$$

Where q_1 and q_2 represent the 10th and 90th quartiles, respectively, computed along each dimension of the dataset.

Once the max_edge_length has been calculated, a V-R complex is constructed around the dataset. For T-PEPT, only the H_0 homology group (connected components) are considered. As the connected components appear and 'die' across the filtration scale the persisting points will represent clusters as other points merge into them. While H_1 and H_2 groups represent deeper insight into the topological features of the dataset, they are not necessary for identifying a distinct number of tracers. Furthermore, by restricting our computations to H_0 we do not perform any additional calculations that are not necessary and streamline our performance. The user can choose to call the 'calculate_persistence' method to visualise the persistence diagram if desired, however, it is not a necessity for reconstructing tracer trajectories from PEPT data.

Fig. 4 shows an example of a real PEPT dataset (in a fluidised bed) containing two tracer trajectories (red and blue) and the corresponding persistence diagram. As we are only concerned with the zeroth homology group, all connected components are 'born' at zero and die off as the max_edge_length is increased. There is one outlying point far from the diagonal at roughly y = 16, corresponding to one final persisting point. However, because of the principles of persistence homology, this single persisting point corresponds to two persisting points merging into a single one, i.e. two clusters merging into one, corresponding to two tracers clusters. The points below the threshold line starting at y = 12.5 represent topological noise and non-persisting features of the dataset. The single point at infinity represents the eventual 'death' of the final cluster in the dataset.

The prolonged presence of the true tracer position in topological persistence diagrams is due to the tracers' design to produce a stable and distinct signal for tracking. This stability ensures that tracers consistently reflect the underlying system's dynamics, resulting in a topological signature that persists across a range of scales in the data analysis. Such a long-lived topological signature is indicative of the tracers' ability to maintain their positional integrity in the face of system perturbations, highlighting their role in accurately mapping the system's stable features and flow dynamics.

In PEPT data, the cutpoints typically form dense regions of points around tracers - these clusters 'absorb' all nearby points as the filtration increases, meaning that we have an evolving topological structure. In the hollow sphere example in Fig. 4 in the main text, once the hole was formed it persisted as a single structure until it 'died'. Conversely, in the case of the two tracers in Fig. 4 we have many points that are merging into the cluster, eventually forming two separate connected components (representing the two tracers), and further merging into a single connected component (the merging of the tracer clusters) with its eventual death at infinity.

2.5 Spatial Clustering

2.5.1 First-Pass Clustering

Before clustering, T-PEPT inserts an artificial cluster outside the field of view (FoV) of the system space, enforcing a characteristic length scale. Further, this artificial cluster gives T-PEPT an example of what a noise cluster should look like in terms of density, size, location, etc., enhancing the algorithm's ability to distinguish between noise and real data clusters. This cluster is assigned a set of labels equal to noise (-1), and post-clustering these labels are removed during analysis to ensure they do not affect the interpretation of the real clusters in the dataset. The true_fraction informs the size of this cluster for calculating the number of points to include [35].

The newly calculated true_fraction is used within the HDBSCAN algorithm, specifically in calculating the min_cluster_size and min_samples. The minimum_cluster_size is defined as the minimum number of points that a cluster has to be considered a cluster; this acts as a threshold for deciding whether a group of densely connected points is a cluster or not. The min_samples parameter is a measure of how conservative the clustering is, where a large value results in more conservative clustering as more points are being declared as noise, with clusters being limited to progressively denser areas [38, 39]. With the true_fraction being dynamically calculated for each sample, the min_cluster_size and min_samples are also dynamically changing for each sample, ensuring that the clustering sensitivity is adjusted according to the level of noise within each sample. Standard HDBSCAN

(a)

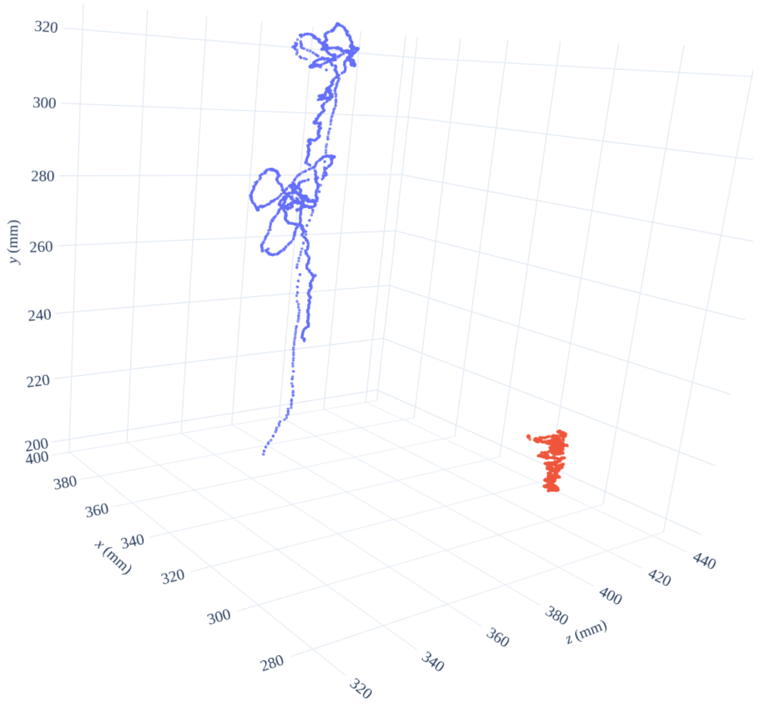

(b)

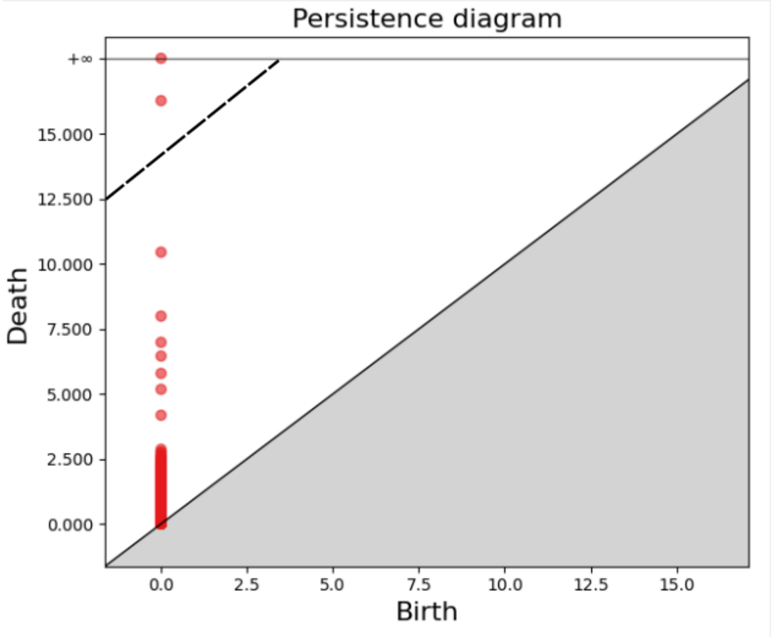

Figure 4: (A) The trajectories of two tracers in a fluidised bed as indicated by the red and blue labelling (plotted using the PEPT library 'PlotlyGrapher()' function). (B) The corresponding persistence diagram of the fluidised bed data. Above a threshold of 12.5, we see two points: (i) the point at y = ∼16 corresponding to two persisting points (the two tracers) merging into a single persisting point, and (ii) the point at infinity corresponding to the eventual collapse ('death') of the single persisting point.

would involve manually choosing the true_fraction value pre-clustering and applying this value to all samples, which may not yield the optimal results for clustering [39].

As mentioned in Section 2.3, HDBSCAN assigns each cluster a unique label (0, 1, 2, ...) and assigns noise a label of -1. These labels are separated and the centroids from each cluster extracted by averaging the x, y, and z positions belonging to all the cutpoints assigned to that cluster. The centre of these cutpoints is then taken as the centroid used to reconstruct the tracer trajectory (Section 2.6), or can be used in a second pass of clustering for more accurate position tracking, as described in Section 2.5.2 [35].

2.5.2 Second-Pass Clustering

Two-pass clustering takes the centroids of each cluster as the centre of the cutpoints, as described in the previous section, from several samples and re-runs the HDBSCAN clustering algorithm. As before, principles from topological data analysis are used to dynamically calculate the true_fraction parameter for each sample, influencing the clustering parameters of HDBSCAN. Nicuşan *et al.* [35] found that a second pass of "reclustering" achieved an increase in tracer position accuracy five-fold, hence why this approach has been applied to T-PEPT.

Obtaining a high spatial and temporal resolution is key for any particle tracking algorithm, and a recent simulation study was conducted with the very goal of analysing the spatial and temporal resolution for a more cost-effective PEPT detector [41]. The second-pass clustering method reduces the temporal resolution for data acquisition due to the use of multiple individual samples, and therefore a larger number of LORs compared to single-pass clustering. However, a 'windowing' technique can be implemented to improve the temporal resolution whereby samples are overlapped. For example, a sample size of 200 with an overlap of 100 means that each sample will consider the last 100 LORs as the first set of LORs within the new sample (e.g. sample1 = [0:200], sample2 = [100:300], sample3 = [200:400], ...). With a large enough window, the decrease in temporal resolution can be removed almost entirely, however, this will act as a smoothing function over the data. It is therefore important to consider the balance between temporal and spatial resolution reduction and over-smoothing, as well as if two-pass clustering is needed for a certain dataset. A slow moving tracer(s) might be better suited to two-pass clustering with large sample size as the smoothing will have a smaller impact on the spatial resolution. Conversely, a fast-moving tracer(s) require high temporal resolution, and therefore the single-pass clustering approach may be more desirable with a small sample size [35].

2.6 Particle Trajectory Reconstruction

The particle trajectories are reconstructed based on the cluster centres, however, there are added challenges when multiple tracers' trajectories intersect or collide as the particle's identity might be lost in such close proximity. The challenge, therefore, is solving how tracers can be correctly identified before, during, and after such occasions. The relationship between a tracer's activity and the number of LORs originating from it is strong; assuming n LORs in a sample, the maximum number of cutpoints to consider is shown in Equation 6. The number of cutpoints scales quadratically with the number of LORs, and as such the differences in activity level between tracers will be amplified when computing the cutpoints: if tracer 1 has an activity of A, and tracer 2 has an activity of 2A, then tracer 2 will have 4-times as many cutpoints as tracer 1. Keeping track of the number of cutpoints is possible when computing the centre of every cluster, and therefore each tracer has a 'signature' that corresponds to the cluster size. In turn, this signature can be used to keep track of each tracer's trajectory [35].

$$max\_cutpoints = \frac{1}{2}(n^2 - n) \tag{6}$$

T-PEPT's use of persistent homology gives each tracer's centroid a 'topological signature' through analysing the spatial and connectivity structures within the data, and subsequently the trajectory. Each tracer trajectory therefore holds information regarding the topology of the paths they follow, and thus adds to the distinguishable features that each tracer has. This trait is particularly useful when dealing with the complex scenarios mentioned above.

It is worth noting that PET detectors have varying sensitivities across their faces, i.e. regions of high and low resolution. In turn, there are numerous factors that can cause problems for tracer detection, such as where the system to be imaged is placed in relation to the detector face(s), the size of the system and tracer(s), the separation between detectors, the activity of the tracer with subsequent 'blinding' effects [31]. Recent studies have looked at the sensitivity of PET detectors for PEPT applications measuring the spatial accuracy across the detectors as a tracer trajectory is moved across the detector's field of view [42].

3 Benchmarking T-PEPT

The recent publication of the "Recent Advances in Positron Emission Particle Tracking: A Comparative Review" [31] was a large collaborative work involving all the major PEPT centres across the world which collectively agreed to developing and directly testing and comparing PEPT algorithms in a rigorous manner via a Standard Testing Framework for PEPT. The following sections show the performance results of T-PEPT over numerous tests to explore the limits of the algorithm. For in-depth details into the tests performed please read this paper [31] and find the data here.

Two overarching cases are needed to test the algorithm rigorously: single-particle tracking and multiple-particle tracking. These will root out the strengths and weaknesses of the algorithm, as well as allow for direct comparison to existing PEPT algorithms. The Birmingham Method is capable of tracking multiple particles, however, due to its functionality and iteration process, it cannot distinguish particle trajectory separations of the same activity. The SBSR algorithm does not yet have multiple particle tracking capabilities.

When changing the activity is not a focus of the test, the tracer(s) activity is set to 10 MBq. This value has been chosen for three core reasons: a) it represents typical activity values used in PEPT experiments, b) it is sufficiently high that the tracer can, theoretically, be tracked in every experimental case, and c) it is sufficiently low enough as to not 'blind' the detectors, resulting in neither detectors experiencing high dead time [31, 43].

All datasets were created using the GEANT4 Application for Tomographic Emission (GATE) toolkit, an open-source simulation software [44]. GATE uses well-validated physics models and emission tomography to model radioactive particle transport through matter, intended for imaging applications such as Single Photon Emission Computed Tomography (SPECT) and Positron Emission Tomography (PET) [45, 44, 46, 47]. GATE has been applied to PEPT, with fully working GATE models of the Philips ADAC Forte dual-headed positron camera and the Siemens ECAT EXACT HR+ ring scanners have been developed by Herald *et al.* [43, 48, 31] for the testing of PEPT algorithms using the "Standard Testing Framework for PEPT" data. The different tests and how T-PEPT compares to the other PEPT algorithms are shown and discussed in the following section.

Details on how the other PEPT algorithms performed before the T-PEPT benchmarks were included are not discussed in detail in this paper but can be found in this paper [31].

3.1 Single Particle Tracking

3.1.1 Minimum Activity Test

The minimum activity an algorithm can track is the most fundamental test that can be performed. This test is performed using a single tracer with a small sample number of LORs covering a wide range of LORs, $N_s \in [10^1, 10^4]$. 10 different N_s repeats are performed for each value, each containing a random set of LORs from a large dataset. Fig. 5 below shows how T-PEPT performs versus the other algorithms.

From the figures, we see that in both detector cases T-PEPT, like the other algorithms, decreases in error with a similar power-law relationship with the number of LORs for both the ADAC and ECAT cases. T-PEPT performs particularly well at low LORs, second only to the Birmingham Method and on par with PEPT-ML in the ADAC case, and outperforming the other algorithms in the ECAT case. T-PEPT performs steadily at all LOR scales, showing its robustness to the sample size, and performing with sub-millimetre resolution with as few as 100 LORs in the ADAC case and with sub-millimetre precision in all instances in the ECAT case. Notably, T-PEPT performs remarkably well in the ADAC case for 1000, 2000, and 10,000 LORs per sample - where each sample contains a fixed number of LORs extracted sequentially from the dataset - compared to PEPT-ML, despite sharing the highest similarity in algorithms.

In a medical setting, being able to perform at such low LOR values would be beneficial to patients undergoing radionuclide treatments as a lower activity source could be used, exposing the patient to a smaller dose of radiation [49]. Further, the fact that T-PEPT performs best in a ring scanner is applicable and beneficial to many medical scanners.

3.1.2 Maximum Velocity Test

The key purpose of this test is to measure how well T-PEPT can track a single fast-moving tracer (fixed at 10 MBq) by following a 'sawtooth' trajectory. With substantial accelerations and discontinuities, the trajectory challenges the algorithm by tracking a tracer with large velocity values ranging from 0.4 ms^{-1} to 20 ms^{-1}. This represents the range of velocities that might be encountered, and exceeded, in real PEPT experiments [31]. The main causes of PEPT error have previously been found to be particle acceleration and gamma-ray scattering, with the PEPT spatial errors increasing linearly with acceleration [50].

(a) (b)

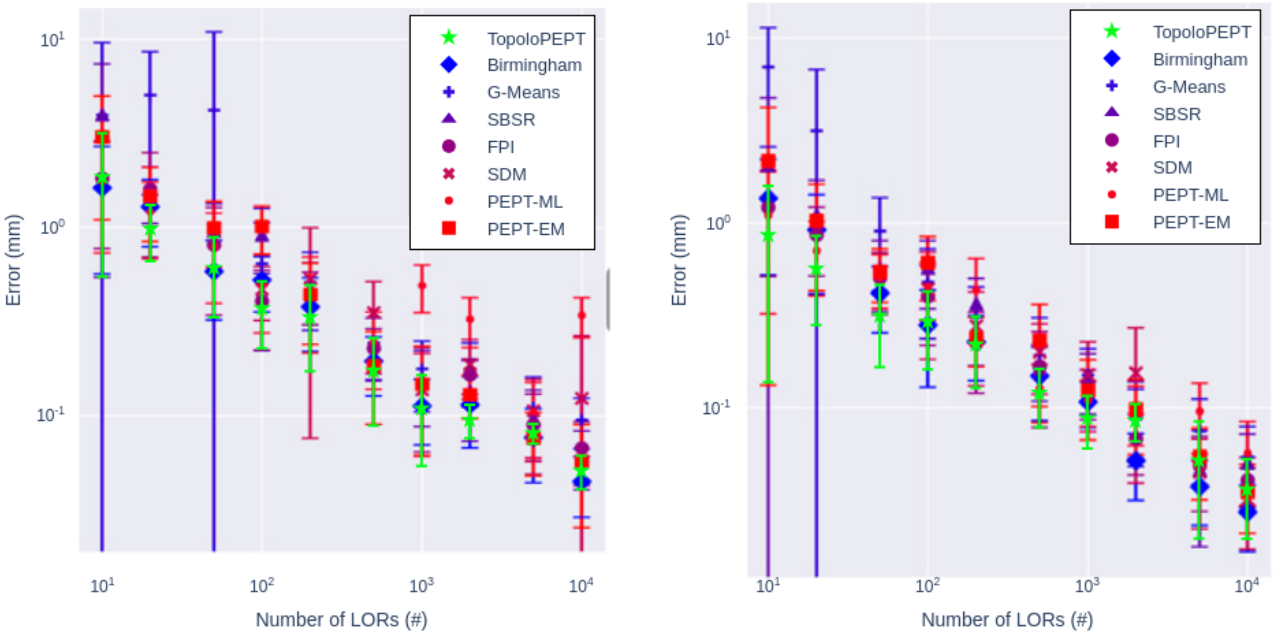

Figure 5: The error, as calculated from equation (20), in PEPT detected positions decreases when more LORs are used. Data is shown for both the ADAC Forté detector geometry (A) and the ECAT EXACT detector geometry (B). The error bars shown represent the standard deviation of the results achieved across multiple repeat tests. This Figure is adapted from Windows-Yule *et al.*, Reports on Progress in Physics, Vol 85, Recent advances in positron emission particle tracking: a comparative review, Copyright IOP Publishing Ltd (2022) [31].

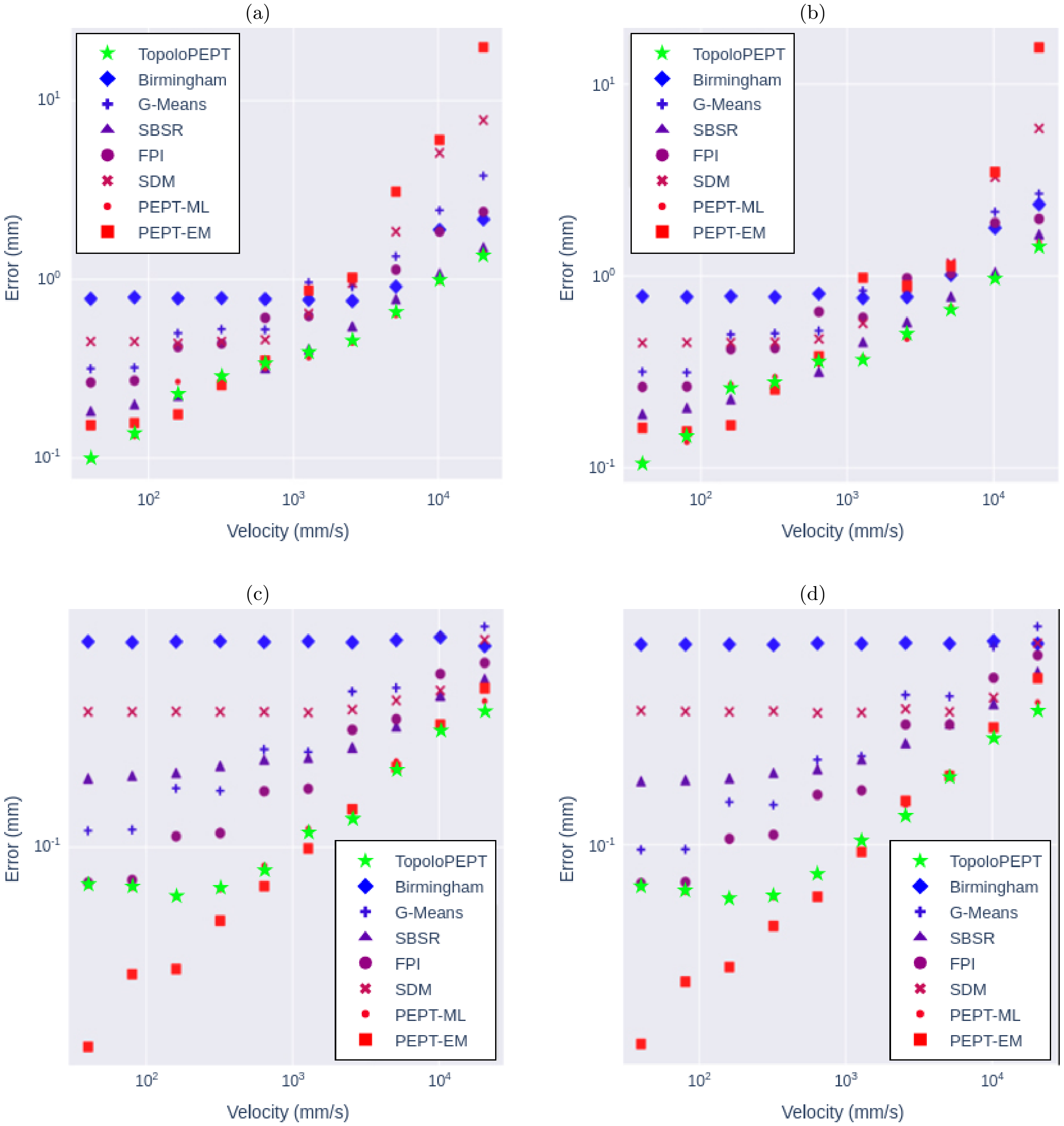

Figure 6: The error in PEPT detected positions increases when tracers move at a higher velocity. (A) Forté detector geometry and movement in X-direction. (B) Forté detector geometry and movement in Z-direction. (C) ECAT detector geometry and movement in X-direction. (D) ECAT detector geometry and movement in Z-direction. This Figure is adapted from Windows-Yule *et al.*, Reports on Progress in Physics, Vol 85, Recent advances in positron emission particle tracking: a comparative review, Copyright IOP Publishing Ltd (2022) [31].

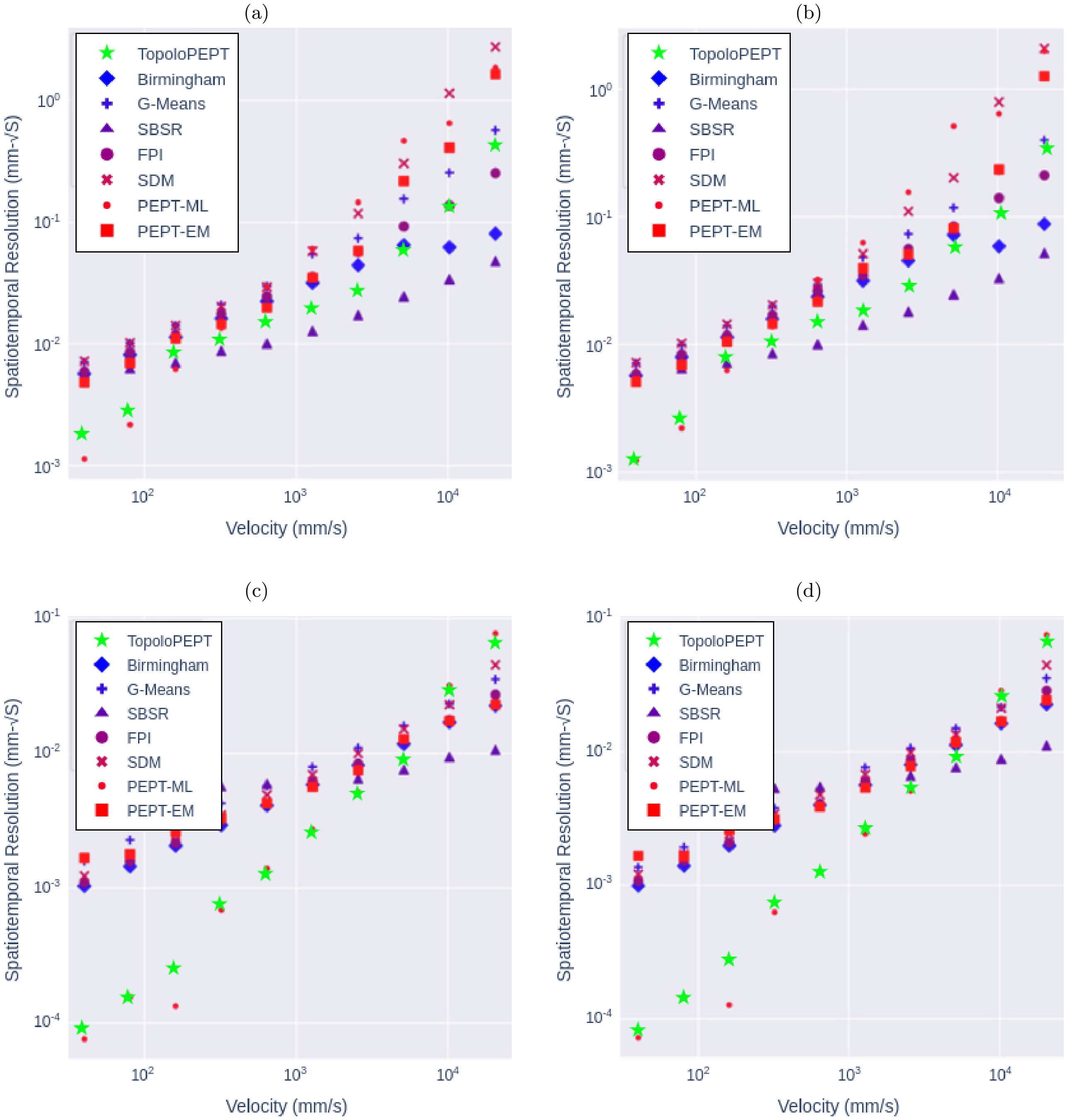

Figure 7: The calculated spatiotemporal resolutions of PEPT detected positions increases when tracers move at a higher velocity. (A) Forté detector geometry and movement in X-direction. (B) Forté detector geometry and movement in Z-direction. (C) ECAT detector geometry and movement in X-direction. (D) ECAT detector geometry and movement in Z-direction. This Figure is adapted from Windows-Yule *et al.*, Reports on Progress in Physics, Vol 85, Recent advances in positron emission particle tracking: a comparative review, Copyright IOP Publishing Ltd (2022) [31].

T-PEPT follows an inverse relationship between velocity and location error for both the ADAC and ECAT detectors when the velocity is in the X-direction, similar to that of PEPT-ML and PEPT-EM, suggesting that T-PEPT is adept at managing the spread of LORs that naturally broadens with accelerated tracer movement. The Birmingham Method, which has high precision with stationary sources, performs relatively steadily but less well with velocity tracking, whereas T-PEPT performs well in both experiments, even with fast-moving tracers. PEPT-ML and SBSR perform very similarly to T-PEPT in these experiments, all performing with sub-millimetre accuracy at all but the highest velocities. In the Z-direction, where the detectors have the lowest spatial resolution, T-PEPT performs better than all algorithms other than PEPT-EM and PEPT-ML, yet slightly outperforms even PEPT-EM at higher velocities.

The spatiotemporal resolution weighs the average location error against the square root of the detection rate of the algorithm. Here, T-PEPT appears to intelligently balance both elements, following an inverse relationship with remarkably high precision at low velocities (along with PEPT-ML), but falling behind the other algorithms at the highest velocities in the Z-direction case.

3.1.3 Scatter Sphere Test Test

This test scrutinises the algorithm's ability to cope with noisy datasets by studying the scatter and attenuation using different materials. A simulated tracer is located inside a 50 mm diameter sphere, with each sub-test changing the scatter media the sphere is comprised of. The materials have attenuation coefficients in the range $\in [0.000111, 1.070]$, from air to BGO. The details of each material can be found in Table 9 from Windows-Yule *et al.* 2021, RoPP [31].

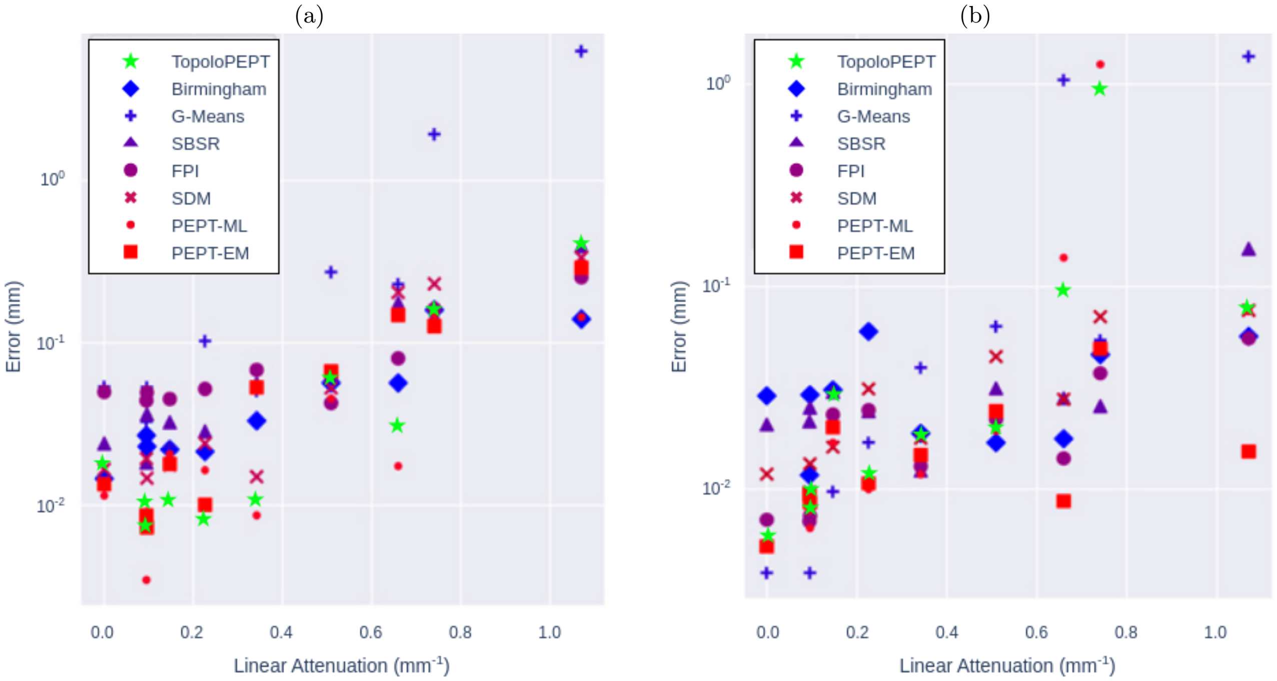

Figure 8: The error in PEPT detected positions increases when more LORs are corrupted by scattering. (A) Forté detector geometry. (B) ECAT detector geometry. This Figure is adapted from Windows-Yule *et al.*, Reports on Progress in Physics, Vol 85, Recent advances in positron emission particle tracking: a comparative review, Copyright IOP Publishing Ltd (2022) [31].

T-PEPT's performance in both detectors reveals sub-millimetre error across all attenuation values. However, it does not consistently perform the best or the worst in either case. This 'middle ground' status indicates it has a well-balanced sensitivity to both low and high levels of scatter, making a safe algorithm choice for performing reliably under a variety of attenuation conditions. PEPT-ML shows the greatest similarity to T-PEPT of all the algorithms, with G-Means being the most dissimilar in the ADAC detector.

3.1.4 Field of View Test

To determine how well the algorithm tracks a particle both in the centre of the field of view (FOV) and towards the edges of the FOV, where the camera's efficiency and sensitivity are notably worse, a simple linear trajectory is simulated. The tracer starts outside of the camera's FOV, then passes through the centre and out the other end [31].

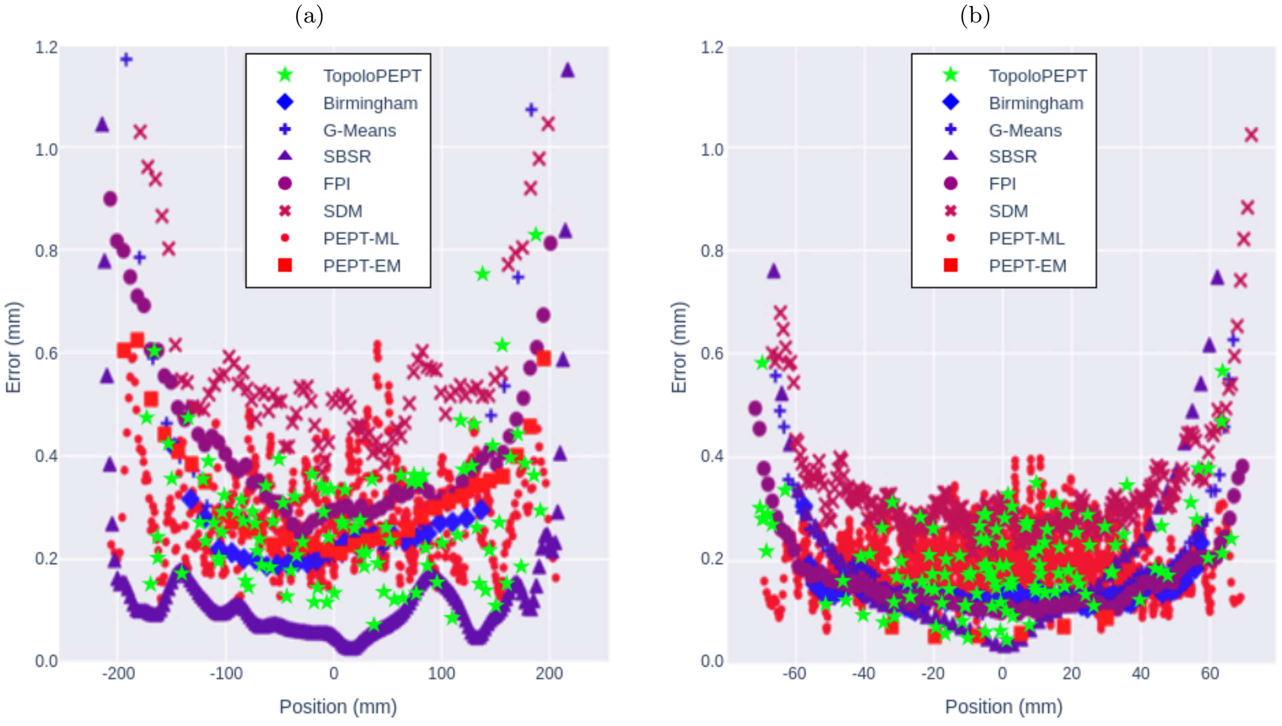

Figure 9: As the tracer nears the edges of the FOV, the error increases. (A) Forté geometry. (B) ECAT geometry. Every fifth detection is plotted and a moving average filter of 20 points is applied to smooth the plotted trajectories for each algorithm. This Figure is adapted from Windows-Yule *et al.*, Reports on Progress in Physics, Vol 85, Recent advances in positron emission particle tracking: a comparative review, Copyright IOP Publishing Ltd (2022) [31].

As is expected, T-PEPT has higher errors toward the edges of the FoV in both the ADAC and ECAT detectors, due to the decrease in detection efficiency and sensitivity in the periphery of the detectors' ranges. T-PEPT appears to exhibit a relatively even spread across the centre of the ADAC detector, with some very low errors on par with SBSR, but also some higher errors along with PEPT-ML, FPR, and SDM. The middle ground suggests a balanced performance without extreme inaccuracies. A similar analysis can be said about the ECAT detector, where the edges show the largest error and the lowest error is at the centre, however, the errors are more variable than in the ADAC, indicating that T-PEPT is more sensitive to this detector configuration.

Despite this variability, the mean error for both the ADAC and ECAT detectors are low, as seen in Fig. 10a, performing comparatively well against the other algorithms. In the ADAC detector, only the Birmingham Method and PEPT-ML perform better, whereas the Birmingham Method, FPI and PEPT-EM perform notably better than T-PEPT in the ECAT detector. Regarding the spatiotemporal resolution, T-PEPT achieves a commendable spatiotemporal resolution, with only PEPT-ML performing better than T-PEPT in both the ADAC and ECAT detectors, suggesting a high degree of accuracy for reconstructing tracer trajectories across both detector's FoV. In particular, the accuracy of the algorithm peaks toward the centre but still performs with an acceptable level of accuracy at the edges.

3.2 Multiple Particle Tracking

3.2.1 Minimum Separation Test

Two simulated 2 mm diameter, 10 MBq tracers are brought into contact from a separation distance, x, of 30 mm down to 2 mm, i.e. to the point of contact of both particles. This tests whether the algorithm can

(a)

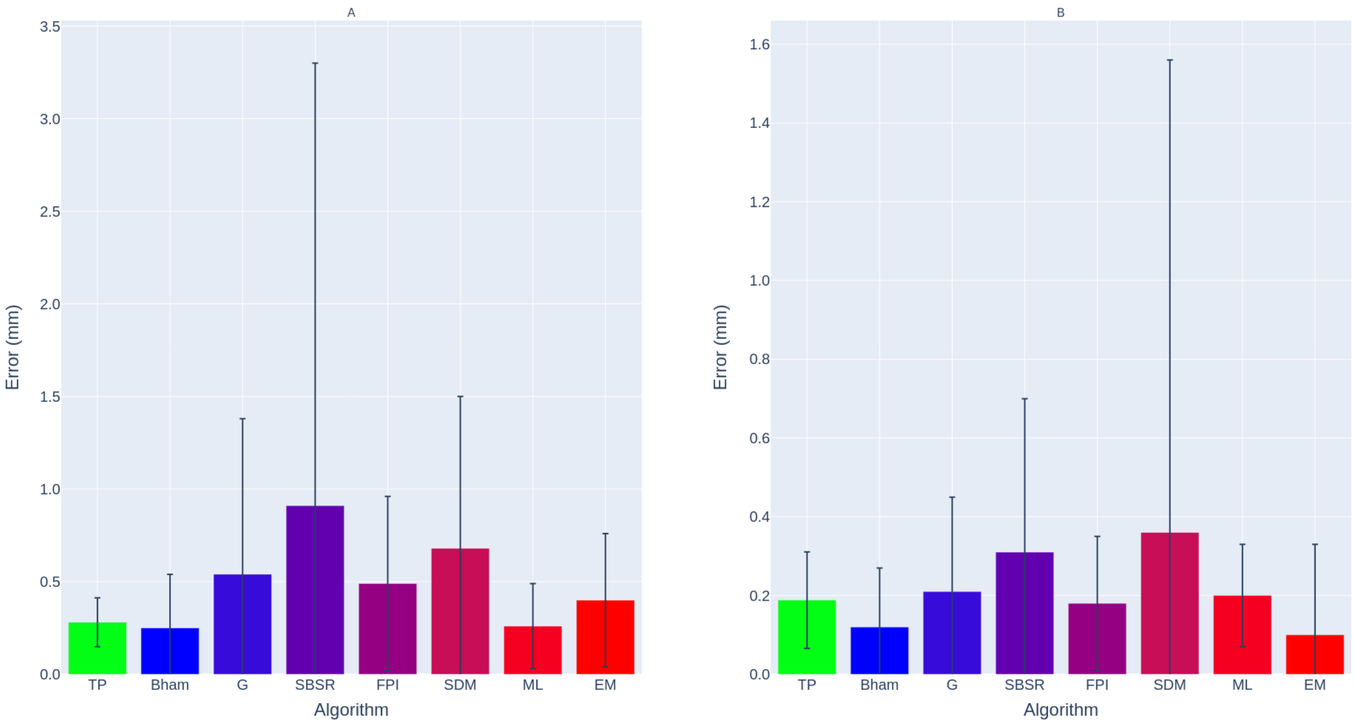

(b)

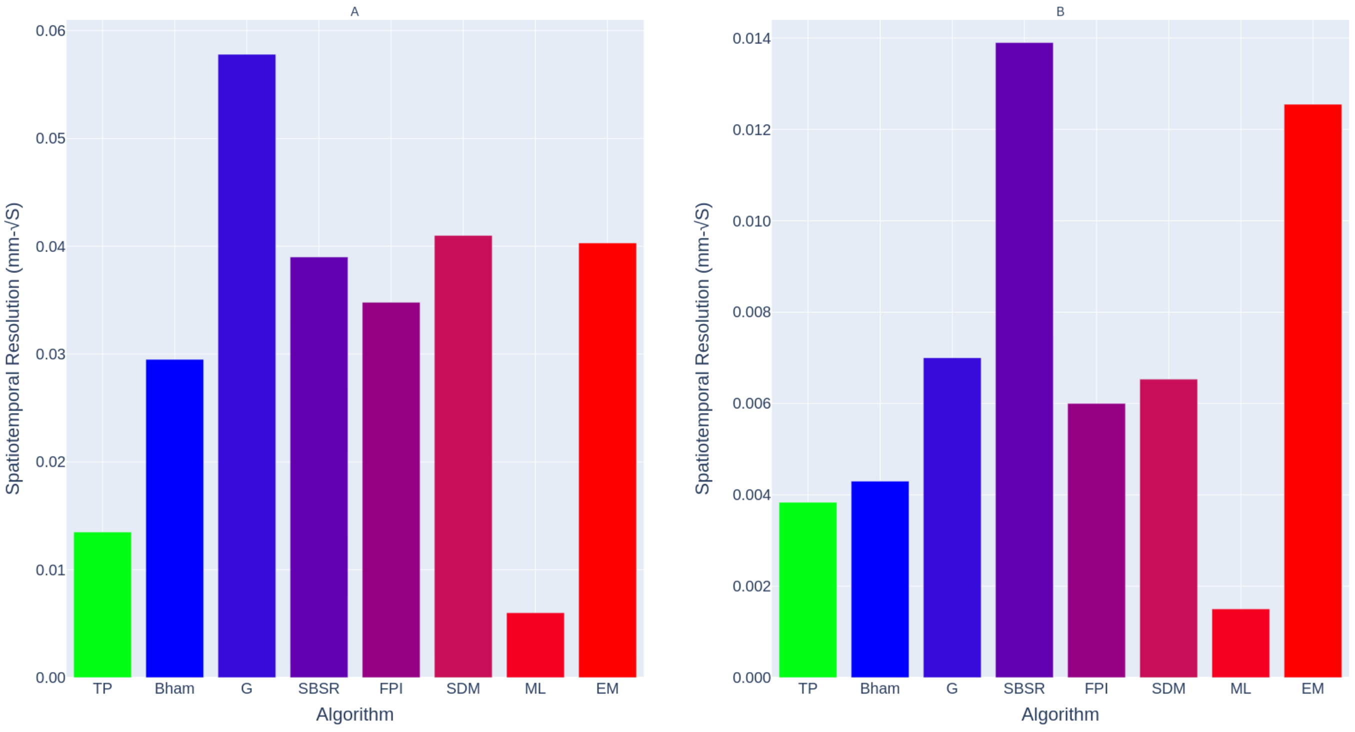

Figure 10: Above: mean errors of each algorithm plotted with error bars denoting the standard deviation of errors. (A) Forté geometry. (B) ECAT geometry. Below: the spatiotemporal resolution returned by each algorithm. (A) Forté geometry. (B) ECAT geometry. Below: the spatiotemporal resolution returned by each algorithm. (A) Forté geometry. (B) ECAT geometry.

successfully separate the tracers, or whether it will falsely merge them into a single location [31].

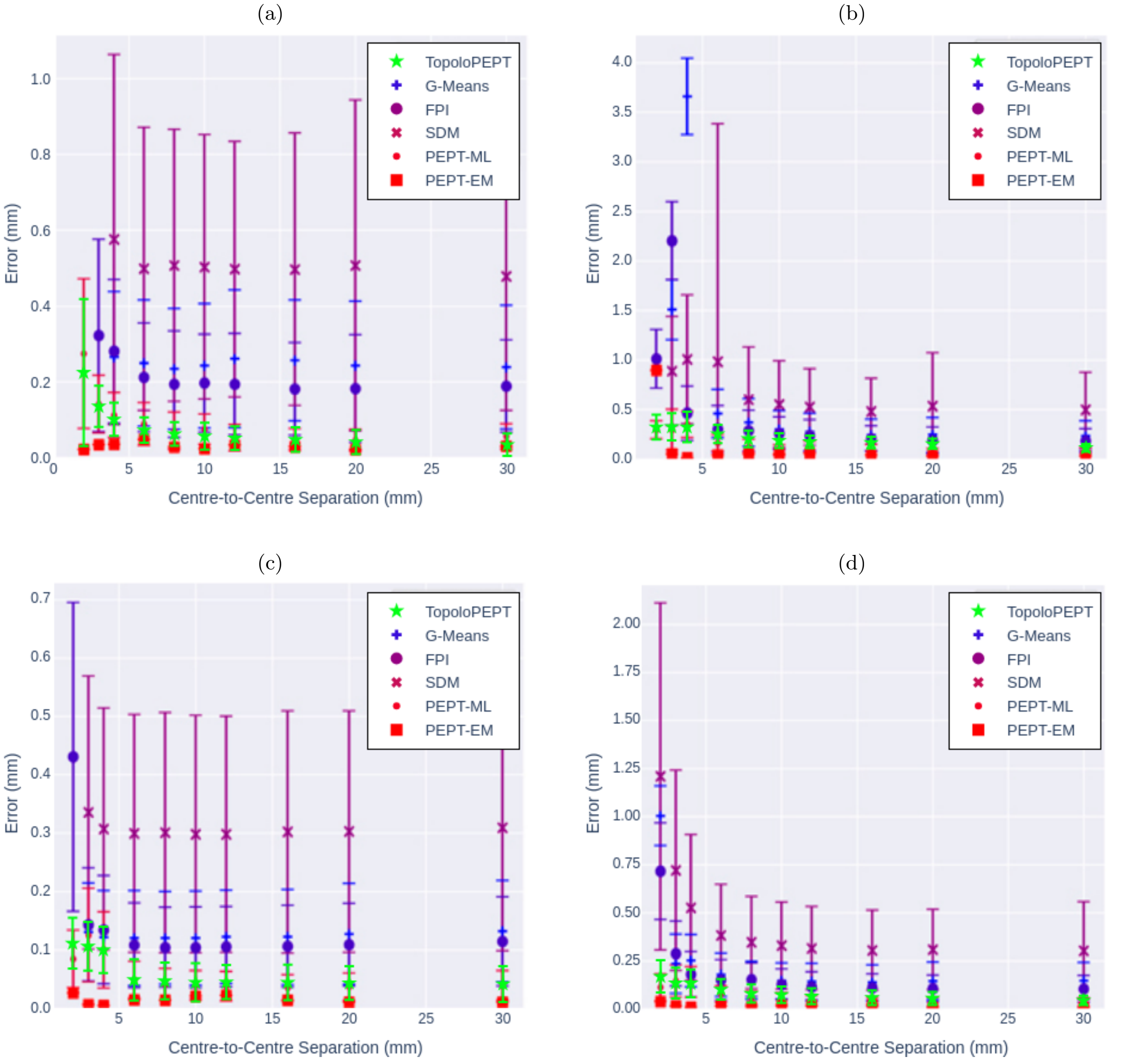

Figure 11: The measured error in PEPT location for the case of two static tracers separated by various centre-to-centre distances ranging from 2 mm (particles in contact) to 30 mm. (A) Forté detector geometry and separation in X-direction. (B) Forté detector geometry and separation in Z-direction. (C) ECAT detector geometry and separation in X-direction. (D) ECAT detector geometry and separation in Z-direction. This Figure is adapted from Windows-Yule *et al.*, Reports on Progress in Physics, Vol 85, Recent advances in positron emission particle tracking: a comparative review, Copyright IOP Publishing Ltd (2022) [31].

T-PEPT successfully separates and correctly locates the two tracers across all distances, even at the extreme 2mm separation, in both detectors, a feat only managed by PEPT-ML, FPI and PEPT-EM. T-PEPT consistently has very low errors close, showing that it can indeed reconstruct tracer locations during collisions as discussed in Section 2.6. T-PEPT performs with sub-millimetre precision in all cases, in both the x- and z-direction, as well as a decrease in standard deviation as the separation increases. PEPT-EM, however, remains to perform the best across all cases bar the ECAT 2 mm separation case, where only PEPT-ML and T-PEPT have smaller errors.

3.2.2 False Positive Test

The goal of this test is to locate 4, 8, and 16 tracers arranged into three different shapes: square, cube, and a tesseract, respectively. These highly symmetric geometries will produce pairs of LORs that will cross over from other tracers' pairs of LORs, designed to test whether the algorithm will be robust against false detection events, or whether they incorrectly interpret the tracers' positions. Every shape has a vertex separation of 10 mm, with one tracer at each vertex, therefore no two particles lie within this distance of each other. 10 mm is both larger than the spatial resolution of the camera and any particle separation of previous tests, thereby not repeating any other abilities [31].

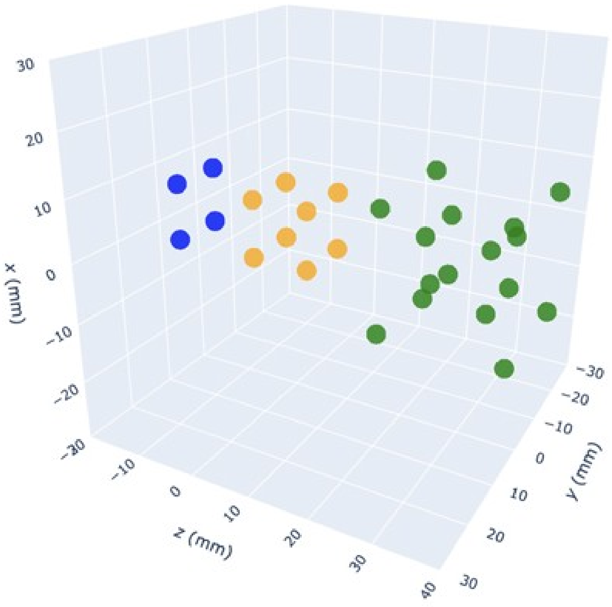

Figure 12: Figure illustrating the positions of groups of tracers for the false positive test. The three tests correspond to groups of 4, 8 and 16 particles arranged, respectively, in a square, a cube and a tesseract (the four-dimensional analogue to a cube). Note that in reality each group of tracers are simulated separately and centred at 0, 0, 0 mm. This Figure is adapted from Windows-Yule *et al.*, Reports on Progress in Physics, Vol 85, Recent advances in positron emission particle tracking: a comparative review, Copyright IOP Publishing Ltd (2022) [31].

As is with the other algorithms, T-PEPT successfully detects all tracers for each scenario, for both detector systems. As one would expect, the error increases with the number of tracers, but within good standing compared to the other algorithms, performing close to, if not with, the lowest error for all numbers of tracers. For the square and cube cases, T-PEPT has sub-millimetre precision, with a competitive error for the tesseract case. Therefore, T-PEPT handles highly symmetrical systems well and is robust against false positives.

3.2.3 Orientation Tracking Test

Three particles are moving at a constant velocity of 0.8 ms^{-1} separated at a fixed distance. The particles follow intersecting paths that map out a sphere of diameter 40 mm. This test looks at an algorithm's ability to locate a tracer at a single point in time, track the tracer over multiple timesteps and reconstruct separated particle trajectories when they cross intersecting points, compared to ground truth data [31].

This test shows that T-PEPT has successfully linked the positions of tracers into unique trajectories, avoiding the common pitfalls of tracer IDs switching during intersections in each dimension, as discussed in Section 2.6. Additionally, the algorithm performs slightly better than its peers in the ADAC detector, with the lowest error bar value, and challenging the likes of G-Means, PEPT-ML and PEPT-EM in the ECAT detector. This precision is indicative of T-PEPT's robust structure. Similar results are evident when looking at the spatiotemporal resolution, with T-PEPT yielding lower values than the G-Means, FPI, SDM and PEPT-EM in both detector cases. This suggests that T-PEPT is capable of tracking multiple linking tracers at a single point in time with a high degree of accuracy.

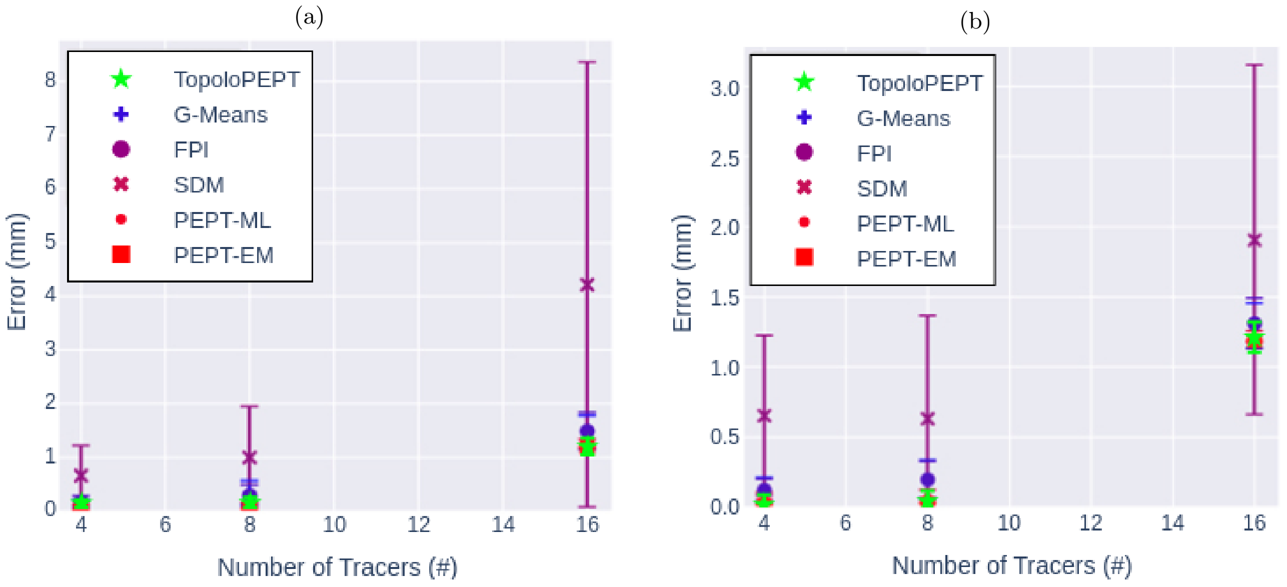

Figure 13: The error in PEPT detected positions for the groups of particles illustrated in Fig. 12. (A) Forté geometry. (B) ECAT geometry. This Figure is adapted from Windows-Yule *et al.*, Reports on Progress in Physics, Vol 85, Recent advances in positron emission particle tracking: a comparative review, Copyright IOP Publishing Ltd (2022) [31].

4 Extra Milling Data

Table 1: Average X-, Y-, and Z-spatial errors for T-PEPT, PEPT-ML, and the Birmingham Method for the 300 RPM case scenarios. Data for which PEPT-ML and the Birmingham Method were unable to locate all 5 tracers successfully are not considered. The number of LORs per sample was 1000 for all three algorithms.

Scenario	Method	No. Tracers Located	Error (mm)		
			X	Y	Z
**Glass Tracer, Aluminium Walls**	T-PEPT	5	0.39(19)	0.76(19)	0.31(19)
	PEPT-ML	5	0.74(20)	1.09(20)	0.62(20)
	BHAM Method	5	1.0(3)	1.7(3)	1.2(3)
**Glass Tracer, Steel Walls**	T-PEPT	5	1.25(25)	1.82(25)	1.37(25)
	PEPT-ML	5	1.6(3)	2.4(3)	1.7(3)
	BHAM Method	5	2.5(5)	3.6(5)	2.8(5)
**Steel Tracer, Steel Walls**	T-PEPT	5	1.67(16)	2.06(16)	1.86(16)
	PEPT-ML	4	3.4(5)	4.5(5)	3.9(5)
	BHAM Method	4	5.4(10)	7.8(10)	6.1(10)

References

[1] Stephen Barr. *Experiments in topology.* Dover Publications, New York, 1989.

[2] G. Carlsson. Topology and data. *Bull. Am. Math. Soc.*, 46:255–308, 2009.

[3] J. L. Kelley. *General topology.* Dover Publications, Inc, Mineola, New York, dover edition edition, 2017.

[4] G. L. Alexanderson. About the cover: Euler and Königsberg's Bridges: A historical view. *Bull. Am. Math. Soc.*, 43:567–574, 2006.

[5] G. W. Dunnington, J. Gray, and F-E. Dohse. *Carl Friedrich Gauss: titan of science.* Mathematical Association of America, Washington, DC, 2004.

(a)

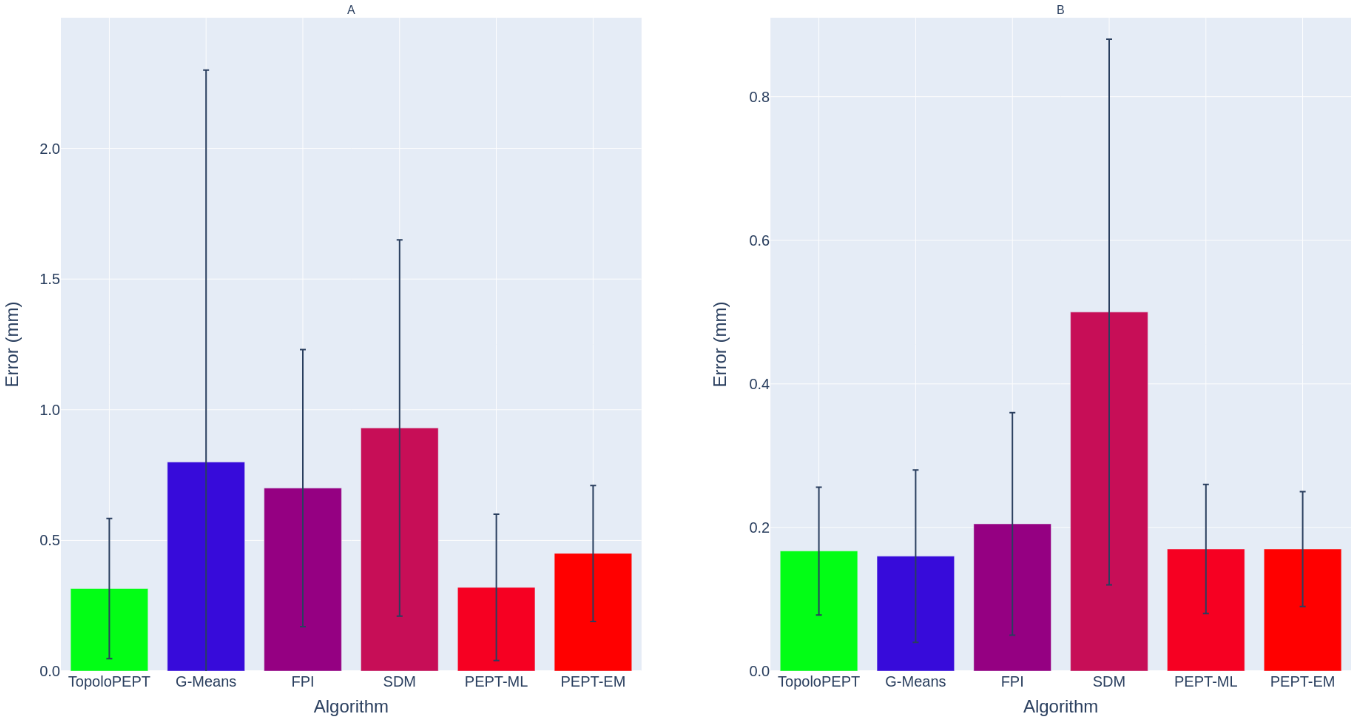

(b)

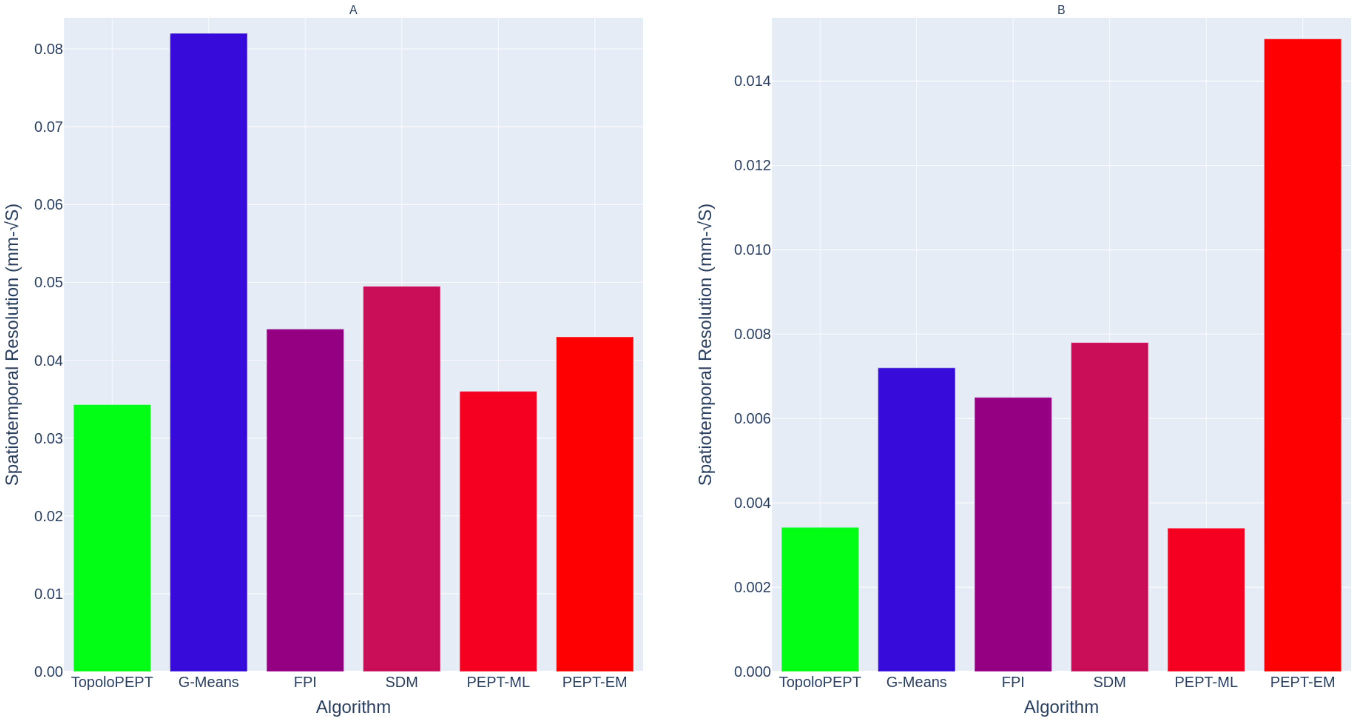

Figure 14: Above: mean error in reconstructed positions of the three tracers (note that all algorithms correctly linked the positions of tracers into unique trajectories). (A) Forté geometry. (B) ECAT geometry. Below: spatiotemporal resolution of the trajectory linking test. (A) Forté geometry. (B) ECAT geometry.

[6] M. A. Armstrong. *Basic topology.* Springer Science & Business Media, 2013.

[7] A. Ganesh, L. Massoulie, and D. Towsley. The effect of network topology on the spread of epidemics. In *Proc. IEEE 24th Annu. Joint Conf. IEEE Comput. Commun. Soc.*, volume 2, pages 1455–1466, Miami, FL, USA, 2005.

[8] D.-W. Zhang, Y.-Q. Zhu, Y. X. Zhao, H. Yan, and S.-L. Zhu. Topological quantum matter with cold atoms. *Adv. Phys.*, 67:253–402, 2018.

[9] L. Panconi, M. Makarova, E. R. Lambert, R. C. May, and D. M. Owen. Topology-based fluorescence image analysis for automated cell identification and segmentation. *J. Biophotonics*, 16, 2023.

[10] S. G. Matthews. Partial metric topology. *Annals of the New York Academy of Sciences*, 728:183–197, 1994.

[11] K. Kuratowski. *Topology: Volume I.* Elsevier Science, Saint Louis, 2014.

[12] J. R. Munkres. *Elements of algebraic topology.* Addison-Wesley, Menlo Park, Calif, 1984.

[13] M. R. Adhikari. *Basic algebraic topology and its applications.* Springer, 2016.

[14] R. Ghrist. Barcodes: The persistent topology of data. *Bull. Am. Math. Soc.*, 45:61–76, 2007.

[15] K. S. Brown. *Cohomology of groups.* Springer Science & Business Media, 2012.

[16] A. Weil. Remarks on the Cohomology of Groups. *Ann. Math.*, 80:149, 1964.

[17] G. W. Whitehead. On Products in Homotopy Groups. *Ann. Math.*, 47:460–475, 1946.

[18] F. Chazal and B. Michel. An Introduction to Topological Data Analysis: Fundamental and Practical Aspects for Data Scientists. *Front. Artif. Intell.*, 4:667963, 2021.

[19] M. Gidea and Y. Katz. Topological data analysis of financial time series: Landscapes of crashes. *Physica A*, 491:820–834, 2018.

[20] A. Zomorodian and G. Carlsson. Computing Persistent Homology. *Discrete Comput. Geom.*, 33:249–274, 2005.

[21] H. Edelsbrunne, D. Letscher, and A. Zomorodian. Topological Persistence and Simplification. *Discrete Comput. Geom.*, 28:511–533, 2002.

[22] J. Kim, J. Shin, F. Chazal, A. Rinaldo, and L. Wasserman. Homotopy Reconstruction via the Cech Complex and the Vietoris-Rips Complex. *ArXiv*, 2019.

[23] S. MacLane. *Homology.* Springer Science & Business Media, 2012.

[24] H. Wu, X. Tao, and Z. C. Zheng. A persistent homology method with modified filtration to characterize the phase trajectory of a turbulent wake flow. *Phys. Fluids*, 33:025118, 2021.

[25] P. Erdös. Graph Theory and Probability. *Can. J. Math.*, 11:34–38, 1959.

[26] K. A. Bryant and B. Karimi. Recognizing patterns in geospatial data using persistent homology: A study of geologic fractures. *Geospatial Data Sci. Tech. Appl.*, 2017.

[27] M. E. Aktas, E. Akbas, and A. E. Fatmaoui. Persistence homology of networks: methods and applications. *Appl. Netw. Sci.*, 4:61, 2019.

[28] B. Olsthoorn. Persistent homology of quantum entanglement. *Phys. Rev. B*, 107:115174, 2023.

[29] F. Chazal, L. J. Guibas, S. Y. Oudot, and P. Skraba. Persistence-based clustering in riemannian manifolds. *Journal of the ACM*, 60:1–38, 2013.

[30] L. Panconi, A. Tansell, A. J. Collins, M. Makarova, and D. M. Owen. Three-dimensional topology-based analysis segments volumetric and spatiotemporal fluorescence microscopy. *Biol. Imaging*, 4, 2024.

[31] C. R. K. Windows-Yule, M. T. Herald, A. L. Nicuşan, C. S. Wiggins, G. Pratx, S. Manger, A. E. Odo, T. Leadbeater, J. Pellico, R. T. M. de Rosales, A. Renaud, I. Govender, L. B. Carasik, A. E. Ruggles, T. Kokalova-Wheldon, J. Seville, and D. J. Parker. Recent advances in positron emission particle tracking: A comparative review. *Reports on Progress in Physics*, November 2021.

[32] C. R. K. Windows-Yule, J. P. K. Seville, A. Ingram, and D. J. Parker. Positron emission particle tracking of granular flows. *Annual Review of Chemical and Biomolecular Engineering*, 11:367–396, 2020.

[33] D. J. Parker, C. J. Broadbent, P. Fowles, M. R. Hawkesworth, and P. McNeil. Positron emission particle tracking - a technique for studying flow within engineering equipment. *Nuclear Instruments and Methods in Physics Research Section A: Accelerators, Spectrometers, Detectors and Associated Equipment*, 326:592–607, 1993.

[34] D. J. Parker, R. N. Forster, P. Fowles, and P. S. Takhar. Positron emission particle tracking using the new Birmingham positron camera. *Nuclear Instruments and Methods in Physics Research Section A: Accelerators, Spectrometers, Detectors and Associated Equipment*, 477:540–545, 2002.

[35] A. L. Nicuşan and C. R. K. Windows-Yule. Positron emission particle tracking using machine learning. *Review of Scientific Instruments*, 91(1):013329, 2020.

[36] P. Ram and K. Sinha. Revisiting kd-tree for nearest neighbor search. In *Proc. 25th ACM SIGKDD Int. Conf. Knowl. Discov. Data Min.*, pages 1378–1388, 2019.

[37] Jean-Daniel Boissonnat and Clément Maria. The Simplex Tree: An Efficient Data Structure for General Simplicial Complexes. *Algorithmica*, 70:406–427, 2014.

[38] R. J. G. B. Campello, D. Moulavi, and J. Sander. Density-based clustering based on hierarchical density estimates. In *Pacific-Asia Conference on Knowledge Discovery and Data Mining*, pages 160–172. Springer, 2013.

[39] L. McInnes, J. Healy, and S. Astels. HDBSCAN: Hierarchical density based clustering. *The Journal of Open Source Software*, 2(11):205, 2017.

[40] A. L. Nicuşan and C. R. K. Windows-Yule. Positron emission particle tracking using machine learning. *Rev. Sci. Instrum.*, 91(1):013329, 2020.

[41] J. Oppotsch, M. Steinke, M. Fritsch, F.-H. Heinsius, T. Held, N. Hilse, V. Scherer, and U. Wiedner. A simulation study on spatial and time resolution for a cost-effective positron emission particle tracking system. *Particuology*, 88:312–322, 2024.

[42] M. T. Herald, J. A. Sykes, D. J. Parker, J. P. K. Seville, T. Wheldon, and C. R. K. Windows-Yule. Improving the accuracy of PEPT algorithms through dynamic parameter optimisation. *Nuclear Instruments and Methods in Physics Research Section A: Accelerators, Spectrometers, Detectors and Associated Equipment*, 1047:167831, 2023.

[43] M. Herald, T. Wheldon, and C. R. K. Windows-Yule. Monte carlo model validation of a detector system used for positron emission particle tracking. *Nuclear Instruments and Methods in Physics Research Section A: Accelerators, Spectrometers, Detectors and Associated Equipment*, 993:165073, 2021.

[44] G. Santin, D. Strul, D. Lazaro, L. Simon, M. Krieguer, M. V. Martins, V. Breton, and C. Morel. GATE: A Geant4-based simulation platform for PET and SPECT integrating movement and time management. *IEEE Transactions on Nuclear Science*, 50(5):1516–1521, 2003.

[45] S. Jan, G. Santin, D. Strul, S. Staelens, K. Assié, D. Autret, S. Avner, R. Barbier, M. Bardiès, P. M. Bloomfield, D. Brasse, V. Breton, P. Bruyndonckx, I. Buvat, A. F. Chatziioannou, Y. Choi, Y. H. Chung, C. Comtat, D. Donnarieix, L. Ferrer, S. J. Glick, C. J. Groiselle, D. Guez, P.-F. Honore, S. Kerhoas-Cavata, A. S. Kirov, V. Kohli, M. Koole, M. Krieguer, D. J. van der. Laan, F. Lamare, G. Largeron, C. Lartizien, D. Lazaro, M. C. Maas, L. Maigne, F. Mayet, F. Melot, C. Merheb, E. Pennacchio, J. Perez, U. Pietrzyk, F. R. Rannou, M. Rey, D. R. Schaart, C. R. Schmidtlein, L. Simon, T. Y. Song, J.-M. Vieira, D. Visvikis, R. V.-D. Walle, E. Wieërs, and C. Morel. GATE: A simulation toolkit for PET and SPECT. *Physics in Medicine and Biology*, 49(19):4543–4561, 2004.

[46] I. Buvat and D. Lazaro. Monte carlo simulations in emission tomography and GATE: An overview. *Nuclear Instruments and Methods in Physics Research Section A: Accelerators, Spectrometers, Detectors and Associated Equipment*, 569:323–329, 2006.

[47] D. Sarrut, M. Bardiès, N. Boussion, N. Freud, S. Jan, J.-M. Létang, G. Loudos, L. Maigne, S. Marcatili, T. Mauxion, P. Papadimitroulas, Y. Perrot, U. Pietrzyk, C. Robert, D. R. Schaart, D. Visvikis, and I. Buvat. A review of the use and potential of the GATE Monte Carlo simulation code for radiation therapy and dosimetry applications: GATE for dosimetry. *Medical Physics*, 41:064301, 2014.

[48] S. Jan, C. Comtat, D. Strul, G. Santin, and R. Trebossen. Monte carlo simulation for the ECAT EXACT HR+ system using GATE. *IEEE Transactions on Nuclear Science*, 52(3):627–633, 2005.

[49] H. Jadvar. Targeted radionuclide therapy: An evolution toward precision cancer treatment. *American Journal of Roentgenology*, 209:277–288, 2017.

[50] M. T. Herald, J. A. Sykes, D. Werner, J. P. K. Seville, and C. R. K. Windows-Yule. DEM2GATE: Combining discrete element method simulation with virtual positron emission particle tracking experiments. *Powder Technology*, 401:117302, 2022.
